# Analysis of NAC Domain Transcription Factor Genes of *Tectona grandis* L.f. Involved in Secondary Cell Wall Deposition

**DOI:** 10.3390/genes11010020

**Published:** 2019-12-23

**Authors:** Fernando Manuel Matias Hurtado, Maísa de Siqueira Pinto, Perla Novais de Oliveira, Diego Mauricio Riaño-Pachón, Laura Beatriz Inocente, Helaine Carrer

**Affiliations:** 1 Department of Biological Sciences, Luiz de Queiroz College of Agriculture (ESALQ), University of São Paulo, Av. Pádua Dias, 11, CP 9, Piracicaba, SP 13418-900, Brazil; fermathur1992@usp.br (F.M.M.H.); maisadesiqueira@gmail.com (M.d.S.P.); perla.oliveira@usp.br (P.N.d.O.);; 2 Computational, Evolutionary and Systems Biology Laboratory, Center for Nuclear Energy in Agriculture (CENA), University of São Paulo. Av. Centenário 303, Piracicaba, SP 13416-000, Brazil; diego.riano@cena.usp.br

**Keywords:** tropical tree, wood formation, secondary growth

## Abstract

NAC proteins are one of the largest families of plant-specific transcription factors (TFs). They regulate diverse complex biological processes, including secondary xylem differentiation and wood formation. Recent genomic and transcriptomic studies of *Tectona grandis* L.f. (teak), one of the most valuable hardwood trees in the world, have allowed identification and analysis of developmental genes. In the present work, *T. grandis* NAC genes were identified and analyzed regarding to their evolution and expression profile during wood formation. We analyzed the recently published *T. grandis* genome, and identified 130 NAC proteins that are coded by 107 gene loci. These proteins were classified into 23 clades of the NAC family, together with *Populus*, *Eucalyptus,* and *Arabidopsis.* Data on transcript expression revealed specific temporal and spatial expression patterns for the majority of teak NAC genes. RT-PCR indicated expression of VND genes (*Tg11g04450-VND2* and *Tg15g08390-VND4*) related to secondary cell wall formation in xylem vessels of 16-year-old juvenile trees. Our findings open a way to further understanding of NAC transcription factor genes in *T. grandis* wood biosynthesis, while they are potentially useful for future studies aiming to improve biomass and wood quality using biotechnological approaches.

## 1. Introduction

NAC proteins are one of the largest families of plant-specific transcription factors (TFs), in which the DNA binding domain is highly conserved. The NAC designation is derived from the *NO APICAL MERISTEM (NAM)* gene from *Petunia hybrida* E. Vilm. and from the *Arabidopsis thaliana* L. genes *ATAF1–2* and *CUP-SHAPED COTYLEDON (CUC2)* [1]. Structures of NAC proteins are commonly divided into two regions, a conserved N-terminal DNA-binding domain and a variable C-terminal transcriptional regulatory region [2]. The N-terminal NAC domains comprise five (A–E) subdomains [2,3,4] located at the N-terminal. Usually, the subdomains contain 150–160 amino acid residues that are implicated in DNA binding, nuclear localization, and formation and localization of homodimers or heterodimers [2]. The C-terminal regions confer regulation of transcriptional activity [3,4]. NAC TFs have been analyzed at the genome-wide level in diverse species such as *Arabidopsis thaliana* L., *Eucalyptus grandis* W.Hill, *Populus trichocarpa* L. (black cottonwood), *Nicotiana tabacum* L. (tobacco), and *Glycine max* (L.) Merr. (soybean) [3,5,6,7,8].

Paleobotanical studies of tracheids resistant to degradation indicate that lignification and secondary cell wall (SCW) differentiation evolved in land plants in the early Devonian (about 415 million years ago) [9]. SCW biosynthesis requires coordinated expression of genes for cellulose, xylan, glucomannan, and lignin biosynthesis. A transcriptional network composed of NAC and MYB transcription factors regulates SCW biosynthetic pathways [10,11]. These closely related TFs function as master transcriptional switches that regulate downstream targets [12]. Thus far, NAC TF genes were identified in all analyzed land plants, whereas eukaryotic algae species lack NAC TFs [13]. Comparative genomic and gene functional analyses have indicated that NAC TFs underwent an expansion in the lineage of vascular plants [13]. Also, tracheid evolution is associated with a burst of structural diversity [14], which is probably related to NAC genes controlling SCW biosynthesis in vascular plants, comprising seedless vascular plants, gymnosperms, and angiosperms [15]. Noticeably, plant species that are structurally less complex contain significantly fewer NAC genes, such as the model bryophyte *Physcomitrella patens* (Hedw.) Mitt. (32 NAC genes), and no NAC genes are found in Chlorophytes (*Ostreococcus lucimarinus* Palenik, *Ostreococcus tauri* C. Courties & M.-J. Chrétiennot-Dinet, *Chlamydomonas reinahrdtii* P.A.Dang.) [16]. Most likely, diversification of NAC genes began with the colonization of terrestrial environments by the ancestors of modern land plants [14]. NAC TFs have been associated with the regulation of a wide range of plant developmental processes acting as transcription activators or repressors. These plant developmental processes include ripening [17], cellular morphogenesis, signaling transduction [18], and establishment of the shoot apical meristem [4], floral organs [19], and lateral roots [20]. They are also involved in the signaling pathway induced during biotic and abiotic stresses [18,21,22], and in the regulation of leaf senescence [23]. As for secondary growth, NAC TFs have been described in the regulation of xylogenesis, fiber development, and secondary cell wall formation [24,25,26,27,28,29,30,31,32].

In Arabidopsis, NAC TFs represent the most upstream regulators in the transcriptional regulatory network that controls wood formation. Master regulators of this development are the VASCULAR NAC DOMAIN (VND1–VND7), NAC SECONDARY WALL THICKENINGs (NST1 and NST2), and SECONDARY WALL NAC DOMAIN TFs (SND1/NST3 and SND2) [10]. These NAC TFs regulate the expression of the MYB genes [11] during SCW formation [12]. Besides, the PROTEIN SOMBRERO (SMB) TF and its two close homologs, BEARSKIN1 (BRN1) and BRN2, are related to the VND/NST TFs in this SCW regulatory network [33]. In this same Arabidopsis regulatory network, the VND-INTERACTING 2 (VNI2) TF is described to negatively regulate VND7 [34]. *Tectona grandis* L.f. (teak), a worldwide attractive tropical tree with valuable wood of high economic importance in industrial forestry, is also a plant species of interest to better understand the SCW biosynthesis and wood formation. Teak’s secondary xylem biosynthesis is a complex molecular process that involves several genes and environmental factors. Recent genomic and transcriptomic analyses of *T. grandis* have allowed to improve the understanding of the xylogenesis role in biomass increase and wood quality [35,36,37,38], even though *T. grandis* NAC transcription factors have not yet been identified. The identification and characterization of teak NAC TFs are fundamental to elucidate their regulation role in vascular tissue differentiation and wood formation, as well as future biotechnological studies aiming to improve biomass and wood quality.

Here, we report a genome-wide identification, phylogenetic profile, gene structure, chromosome localization, phylogenetic relationship, and expression profiles of *T. grandis* NAC genes related to wood formation. In addition, tissue-specific expression patterns of 13 teak NAC TF genes homologous to Arabidopsis in their response to wood formation were investigated by quantitative real-time RT-PCR (RT-qPCR). The data represent a step forward to the understanding of plant NAC transcription factors and the regulation of secondary cell wall biosynthesis and wood formation in *T. grandis*.

## 2. Materials and Methods

### 2.1. Database Search and Phylogenetic Profiles

The *Tectona grandis* NAC genes were compared to the model species *Eucalyptus grandis*, *Populus trichocarpa,* and *Arabidopsis thaliana* in which NAC genes were already identified and analyzed [3,5,6]. Deduced proteomes of *Eucalyptus grandis* [39] and *Populus trichocarpa* [40] were obtained from Phytozome v12, and The Arabidopsis Portal [41] was used for *A. thaliana* proteins. As for *T. grandis,* the proteins were accessed in the Dryad database [38]. We considered that any protein carrying the NAM domain was a member of the NAC family of TFs. The NAM domain was searched and identified using the profile Hidden Markov Model for this domain from PFAM (Accession Number PF02365). This profile was downloaded from Pfam Database (http://pfam.sanger.ac.uk/) and a hmm-search was performed with the HMMer software v3.2.1 (Cambridge, Massachusetts, United States) ‘hmmscan’. Hit scores higher than 21.2 (gathering cutoff from PFAM specific for the NAM model) were considered true positives and selected for further analyses. A diagram of the phylogenetic relationship among teak and 28 species was constructed with the tree generator PhyloT (http://phylot.biobyte.de/index.html) based on the NCBI taxonomy IDs. The APGIII classification system was used to manually indicate the phylogenetic orders of the flowering plants species in the tree [42]. As for *Clorophyta*, *Bryophyta,* and *Licophyta*, the classification was done according to Novikiv and Barabas-Krasni [43].

### 2.2. Phylogenetic Analysis of NAC Transcriptional Factors

Non-redundant peptide sequences of NAC domain proteins (Pfam ID: NAM; PF02365) from *Tectona grandis* (116), *Populus trichocarpa* (196), *Arabidopsis thaliana* (153), and *Eucalyptus grandis* (190), previously identified (Section 2.1), were aligned using MAFFT v7.407 in the auto mode [44]. Poorly aligning regions from the multiple sequence alignment were removed using TrimAl v1.4, in the “automated1” mode [45]. Phylogenetic inference under the Maximum Likelihood approach was carried out with IQ-Tree v1.6.9 [46], with the options “”-m MFP -st AA -seed 12345 -lmap 65400 -alrt 1000 -bb 1000”. The best evolutionary model for phylogenetic inference was also estimated within IQ-Tree with the option -m MFP (Model Finder Plus), by computing the log-likelihoods for many different evolutionary models against an initial parsimony tree and choosing the model that minimizes the Bayesian information criterion (BIC) [47]. Branch support was assessed with the Shimodaira–Hasegawa (SH)-like approximate likelihood ratio test [46] and ultrafast bootstrap [48], both with 1000 replicates. The multi-species phylogenetic tree of NAC TFs was reconciled with the species tree, using Notung 2.9 in order to infer the most likely root and the history of gene duplications in the NAC gene family [49]. Briefly, the species tree–gene tree reconciliation process tries to account for the differences between the species tree and the gene tree, with the gene-level processes of gene duplication and gene losses.

### 2.3. Chromosomal Location

The teak NAC genes were mapped on chromosomes in accordance with the whole genome of this species (https://datadryad.org/stash/dataset/doi:10.5061/dryad.77b2422) [38]. Of the 18 teak chromosomes, 17 near-complete pseudomolecules were generated with one chromosome present as two chromosome arm scaffolds [38]. Chromosomal locations of the identified teak NAC genes were extracted from the general feature format (GFF) file provided with the genome sequence and visualized in the Integrative Genomics Viewer (IGV v2.7.0) [50]. Note that for the purpose of the visualization of NAC genes on teak chromosomes, the pseudomolecules 18 and 19 were joined into a single chromosome.

### 2.4. Gene Structure and Conserved Motifs

Exon–intron structures of *T. grandis* NAC genes were analyzed and illustrated with the Gene Structure Display Server (GSDS) (http://gsds.gao-lab.org/index.php) by comparison of coding sequence (CDS) regions with genomic DNA sequences of this species [38]. The software MEME v5.0.5 (MEME—http://meme-suite.org/index.html) was employed for the detection of conserved motifs with the following parameters: Distribution of motif occurrences, zero or one per sequence; minimum width, 6, maximum width, 50; maximum number of motifs, 10; and optimum motif width, ≥6 and ≤116 [51]. The subcellular localization of the identified NAC proteins was predicted by using an online analysis tool from Molecular Bioinformatics Center v2.5 (http://cello.life.nctu.edu.tw/) and BUSCA (http://busca.biocomp.unibo.it)

### 2.5. In Silico Gene Expression Profiling

The RAW RNASeq data from *T. grandis* were accessed in the Sequence Read Archive (SRA-NCBI) under the accession number SRP059970. These data were previously generated by our group [36] and comprise various plant tissues in three plant developmental stages. The teak RAW RNASeq data were processed with BBDuk2 [52] in order to remove low-quality regions from the reads, remainders of adapter sequences, and ribosomal RNA. Salmon v1.0.0 [53] was employed to estimate expression values, as transcripts per million (TPM), using as reference the predicted cDNAs from the *T. grandis* genome [38]. Expression values were imported into R [54] with the tximport package [55] and summarized to gene level. Gene expression values for each gene were transformed using *Z*-scores, i.e., the expression value of a gene in a given condition was subtracted from the average gene expression and expressed as number of standard deviation from the mean, and then visualized as heatmaps using the pheatmap package.

### 2.6. Expression Analysis of Marker Genes for Secondary Cell Wall Formation by RT-qPCR

Expression patterns of genes involved in wood formation were analyzed in samples of teak stem collected from 4-year-old plants grown in the greenhouse, and sapwood samples collected from 16- and 64-year-old trees grown in a field in Piracicaba, São Paulo State, Brazil (Latitude: 22°42′23″S, Longitude: 47°37′7″W, 650 m above sea level). This is the same population of trees used for the previously reported transcriptional profile mentioned in Section 2.5 [36]. In this expression analysis, new tissue samples were collected with a Pressler borer at DBH, 4 years apart of the first sampling [36]. Frozen tissue samples (0.6 g) were ground to a fine powder in liquid nitrogen using a sterilized mortar and pestle. Total RNA was extracted following the Trizol (Invitrogen, Carlsbad, California, USA) protocol. RNA was quantified in a NanoDrop 2000 spectrophotometer (Thermo Scientific, Carlsbad, California, USA) and RNA integrity was examined by gel electrophoresis. Total RNA was treated with DNAse I (Promega, Madison, Wisconsin, USA) and then used for cDNA synthesis using SuperScript™ III First-Strand Synthesis System for RT-PCR (Invitrogen, Carlsbad, California, USA), according to the manufacturer’s instructions. Quantitative RT-PCR (qPCR) reactions were conducted in 12.5 µL total volume using a Platinum Sybr Green Supermix (Invitrogen, Carlsbad, California, USA), and ran in an ABI 7500 qPCR thermocycler (Applied Biosystems, Foster City, California, USA). Expression data were normalized using the 2-ΔCt method. The constitutive *Elongation Factor-1 alpha* (*EF-1 alpha*) housekeeping gene was used as internal control [56]. We used three biological repetitions and two technical repetitions. The statistical analysis was performed using Prism v8 software (GraphPad, San Diego, California, USA). Normality of data was confirmed by the Shapiro–Wilk test, which indicated that the data follow normal distribution. Statistical significance was determined by one-way ANOVA, with Tukey post-hoc analysis (*p* > 0.1). The analyzed genes are key regulators of SCW formation in Arabidopsis (Table S1).

## 3. Results and Discussion

### 3.1. Identification and Evolution Analyses of NAC Family Transcription Factors

The analysis of teak NAC TF encoding genes identified 130 NAC proteins that are coded by 107 loci. We analyzed, with CD-HIT, the 130 teak NAC proteins and identified 14 that were 100% identical to another protein in the original set. Thus, we generated a set of non-redundant proteins (at 100% identity), which has 116 proteins, and used that set for phylogenetic analysis. This procedure was also carried out for each species in the phylogenetic analyses. Each gene was annotated as TgNAC0XX, where Tg refers to the initials of *Tectona grandis* and XX refers to the position of the gene in the chromosomes. Detailed information of teak’s NAC family genes, including gene locus, given code, accession numbers, and similarities to their Arabidopsis orthologues, is listed in Table S2.

NAC TF genes have been identified in all analyzed land plants, but so far, they have not been found in eukaryotic algae species [14] (Figure S1). The NAC multigene family is highly variable among plant species. Presumably, NAC TFs first appeared in bryophytes such as *Physcomitrella patens* and, most likely, they were responsible for the expansion of the NAC genes named VNS (VND-NST/SND-SMB-related proteins) in land plants [13,57]. *Populus* (Rosideae) contains large numbers of NAC genes in a relatively small genome size compared to *Eucalyptus grandis* and *Glycine max*, both containing large numbers of NAC genes in larger genome sizes. Expansion of NAC genes in various plant species is probably the result of multiple events of gene duplication. In the *Populus* lineage, for example, whole genome duplication and multiple segmental and tandem duplication events may have contributed to the expansion of the NAC family [40]. As for *T. grandis*, the small number of duplication events could be one of the reasons for the relatively small number of identified NAC genes (107). As depicted in Figure S2, only 36 gene duplication events were inferred for *T. grandis*, whereas they were 77 in Arabidopsis, 114 in Eucalyptus, and 98 in *Populus*. It is also curious that only 31 NAC genes were found in the coniferous *Pinus taeda*, a species with a large genome, possibly due to the high content of pseudogenes in conifer genomes [58]. Besides, only two genes of the VASCULAR NAC DOMAIN (VND) family were identified in *Pinus taeda*. This finding could be interpreted as evidence of the importance of co-option and expansion of the VND gene family during the evolution of angiosperms [59].

### 3.2. Phylogenetic Analysis of the NAC Gene Family

**Figure 1:**
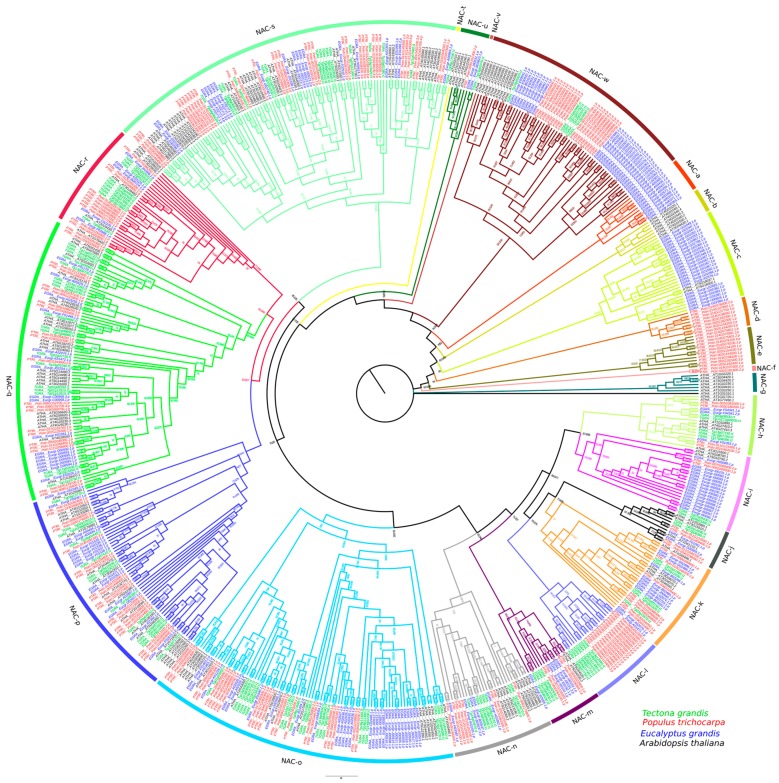
Maximum-likelihood phylogenetic tree rooted of NAC domain proteins of *T. grandis*, *Populus*, Arabidopsis, and *Eucalyptus*. The NAC family proteins were clustered in 23 distinct clades (NAC-a–NAC-w). Members of *T. grandis* (116), *Populus* (196), Arabidopsis (153), and *Eucalyptus* (190) NAC protein family were denoted with green, brown, black, and blue letters, respectively. Bootstrap values are described in the branch points.

Evolutionary relationships were examined among non-redundant NAC protein sequences of *T.grandis* (116), *Populus trichocarpa* (196), *Eucalyptus grandis* (190), and *Arabidopsis thaliana* (153). A rooted tree was constructed and maximum-likelihood algorithm separated the NAC family proteins into 23 distinct clades (NAC-a–NAC-w), as represented in Figure 1. *T. grandis* NAC proteins were identified in 14 of the 23 clades (NAC-h–NAC-s, NAC-u, and NAC-w).

Clade NAC-s clustered the largest group of conserved genes that consisted of *T. grandis* (16 genes), *Eucalyptus* (19 genes), *Populus* (34 genes), and Arabidopsis (34 genes) sequences (Figure 1). Clades NAC-d, -e, and -f comprised exclusively *Populus* NAC genes and that is consistent with the evidence of major gene duplication events in this species [40]. In general, NAC members were interspersed among the majority of the clades, which indicates expansion of NAC genes previously to the evolutionary divergence of *T. grandis*, *Populus*, Arabidopsis, and *Eucalyptus*.

Noticeably, NAC genes with the same functions tended to fall into a same clade, as previously reported [60]. For instance, clade NAC-p grouped SCW biosynthesis genes involved in xylem vessels biosynthesis (*VND4* (*Tg15g08390, Tg16g07170*), *VND1* (*Tg09g04510, TgUn296g00020*), *VND7* (*Tg03g10560, Tg03g10970, Tg18g00740*)) and in biosynthesis of fibers (*NSTI* (*Tg05g19210, Tg05g19290, Tg11g07410*) and *SMB* (*Tg02g10510*)). As for VNI genes, both VNI1 (*Tg09g02550*) and VNI2 (*Tg10g07670*, *Tg11g14670*, *Tg16g09090*, *Tg15g04300*, *Tg16g09080*) genes were located in clade NAC-n, with the exception of *Tg12g02970* (VNI2), that was placed in clade NAC-w. The SND2 genes (*Tg02g15550*, *Tg17g02630*) were clustered in clade NAC-q (Figure 1).

### 3.3. Gene Structure and Conserved Motifs of Teak NAC Genes

**Figure 2:**
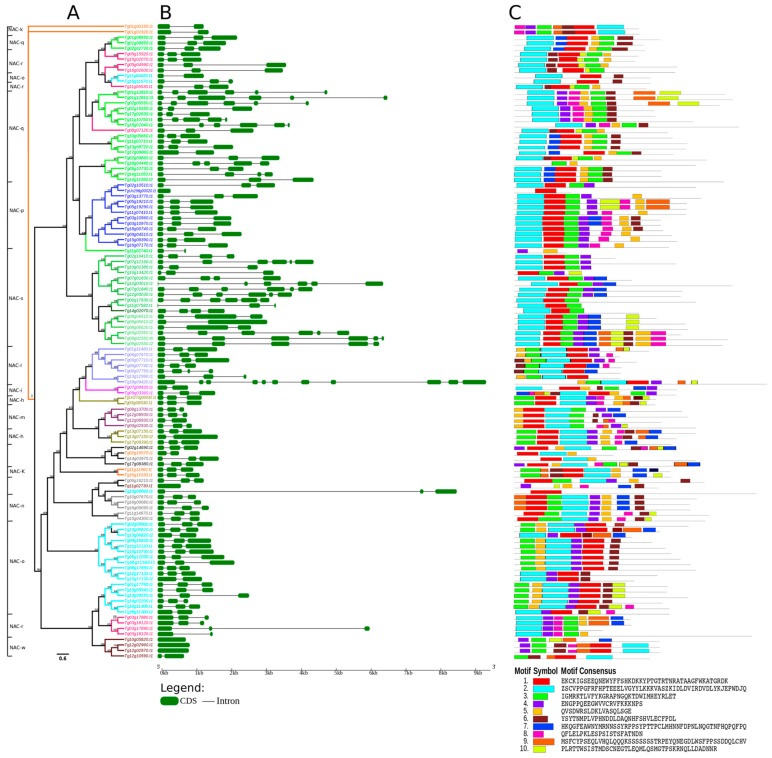
Phylogenetic relationship, gene structure, and motif composition of *T. grandis* NAC genes. (**A**) Maximum-likelihood phylogenetic tree rooted of 116 teak NAC proteins was constructed using Q-Tree v1.6.9 and ultrafast bootstrap, with 1000 replicates. (**B**) Exon–intron structure represented in kilobase (kb) scale of 107 TgNAC genes. (**C**) Schematic representation of the conserved motifs in the NAC proteins from teak. Green boxes and black lines represent exons and introns, respectively.

Variations in gene and protein structure are the basis of evolution in multigene families [61]. A structural analysis of teak NAC genes and proteins was performed in order to gain information on structural diversity among them. In the phylogenetic tree, the analyzed proteins were clustered according to the presence of conserved motifs, as shown in Figure 2A. The color of protein names refers to the clades in Figure 1. Exon–intron structure of TgNAC coding sequences was also analyzed individually (Figure 2B). NAC members of a same clade shared similar gene length and exon–intron structure.

In total, 10 divergent motifs were localized and named as motifs 1–10 (Figure 2C, Table S3). The motifs 1–3 and 5–7 were recognized as NAC subdomains in *Eucalyptus*, as described previously [5,62]. Although motifs 4 and 8 have already been described in *Eucalyptus* [5] and *Populus* [6], respectively, they were not recognized as NAC. The motifs 9 and 10 were not described until this study. As expected, most phylogeny-based groups displayed common motifs with the same alignment and position. Thus, NAC proteins with similar gene structures and motifs tended to cluster in the same clade. Clade NAC-q contains the proteins VNS, VNDs (VND1, VND4, and VND7), NST1, and SMB that have very conserved structures (Figure 2A,C). The gene SMB is among the groups of NAC genes present in early land plants, such as the bryophyte *Physcomitrella patens* [63]. Proteins that were clustered in the clades NAC-o, NAC-s, and NAC-q also shared high similarity in their structures, as shown in Figure 2C. The shortest teak TgNAC gene (TgUn720g00010) is 544 bp long, and the two longest genes (Tg12g03010 and Tg09g02550) are 6 kb in size. Most *T. grandis* genes contained introns in their sequences, except for *Tg10g05820*, *Tg11g02730*, *Tg12g02970,* and *TgUn296g00020* (Figure 2B). The subcellular localization of the identified NAC proteins was predicted by using the online analysis tools Molecular Bioinformatics Center v2.5 (http://cello.life.nctu.edu.tw/) and BUSCA (http://busca.biocomp.unibo.it). *T. grandis* NAC proteins were located in the nucleus, cytoplasm, chloroplast, plasma membrane, endomembrane system, and mitochondria. Such subcellular localization is similar to those of various plant species, such as *Zea mays* and *Cucumin sativus,* in which NAC TFs are also located in diverse cell compartments (Table S4) [64,65].

### 3.4. Chromosomal Location

**Figure 3:**
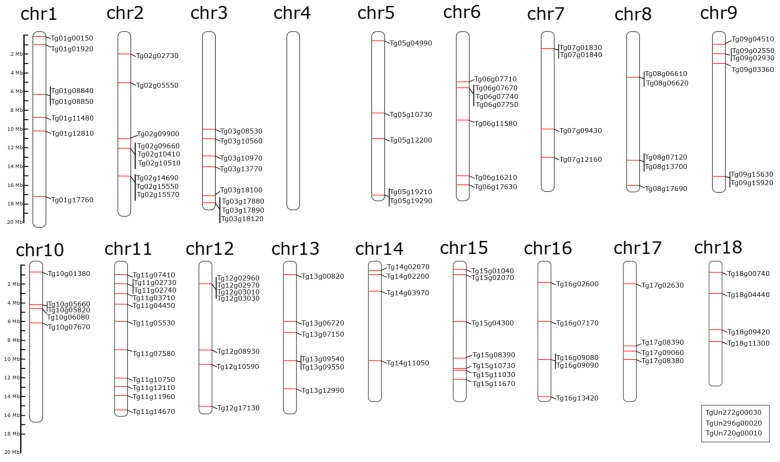
Location and distribution of 107 TgNAC genes on 18 *T. grandis* chromosomes. The box at the bottom right-hand side represents three TgNAC genes that were not placed within any of the chromosomes and were present in orphan scaffolds. TgNAC genes were mapped according to their genomic position in the pseudomolecules (Table S5). The scale on the left-hand side is represented by megabase (Mb).

Of the 18 teak chromosomes, 17 were generated as near-complete pseudomolecules, and one of them as two chromosome arm scaffolds [38]. Integrative Genomics Viewer (IGV v2.7.0 - Cambridge, Massachusetts, USA) was used for assembling the 18 chromosomes (Chr) and to localize the 107 NAC genes, as represented in Figure 3. Three of the 107 genes (*TgUn272g00030*, *TgUn296g00020*, *TgUn720g00010*), identified by using the published *T. grandis* genome [38], were located in scaffolds that have not been placed within a chromosome; therefore, they were represented in a box at the bottom right-hand corner of Figure 3. The largest number of TgNAC genes was located on Chr 11 and consisted of 11 TgNAC genes. Other TgNAC genes were distributed among 16 of the remaining chromosomes, as follows: 9 on Chr2, 8 on Chr3, 7 on chromosomes 1, 6, 12, and 15, and in lower numbers among the other teak chromosomes. No NAC gene was identified on Chr4. Likewise, unequal distributions of NAC TF genes across chromosomes and highest gene density in distal regions have been reported in barley and wheat [66,67].

Many of the teak NAC genes were located adjacent to each other (less than 10,000 bp apart), as, for instance, *Tg01g08840* and *Tg01g08850* on Chr1, *Tg03g17880* and *Tg03g17890* on Chr3, *Tg06g07740* and *Tg06g07750* on Chr6, *Tg07g01830* and *Tg07g01840* on Chr7, *Tg12g02960* and *Tg12g02970* on Chr12, and *Tg16g09080* and *Tg16g09090* on Chr16 (Figure 3, Table S5). Most likely, these closely adjacent genes could have resulted from duplication events. In wheat, single loci are found more frequently in proximal regions of the chromosomes, while gene duplications often occur in the distal regions [67].

### 3.5. In Silico Gene Expression Profiles of Teak NAC Genes

**Figure 4:**
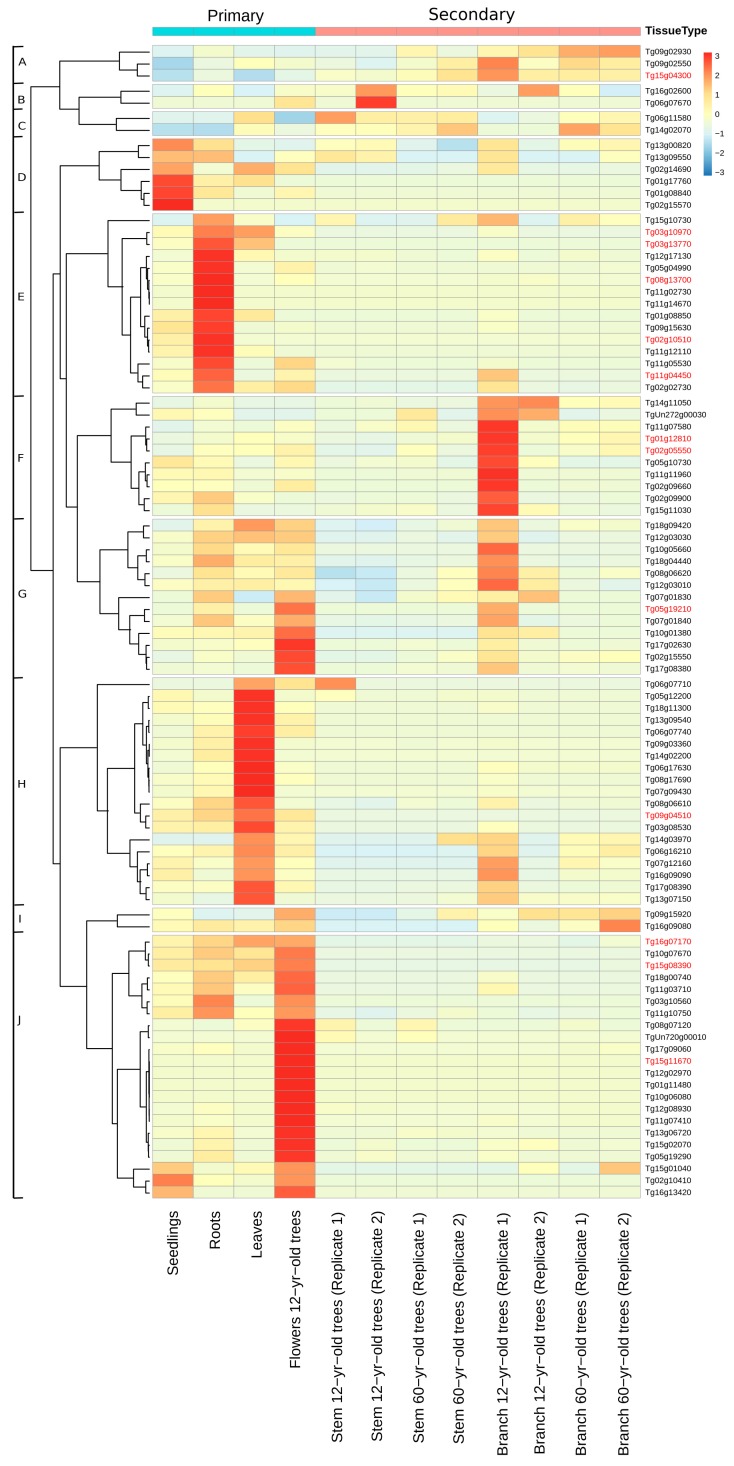
Hierarchical clustering of expression profiles of TgNAC genes in diverse *T. grandis* tissues. The 94 genes were clustered into 10 groups (**A**–**J**). Plant tissues (branch of 12- and 60-year-old trees; stem of 12- and 60-year-old trees, flowers, leaves, roots, and seedlings) were clustered according to the type tissue. The color scale on the left-hand side represents transcript per million (TPM). Gene expression values for each gene were transformed using *Z*-scores. Higher expression levels are represented in red, and lower expression levels are denoted in blue (Table S6). The TgNAC TF genes marked in red were selected for expression analysis.

Expression profiles of 107 TgNAC genes were assessed in order to gain insights into their transcript accumulations. Transcript abundance analyses were carried out for diverse tissues of teak, including primary (leaves, flowers, roots, seedling) and secondary (branch and stem secondary xylem of 12- and 60-year-old plants) tissues [36]. Of the 107 analyzed TgNAC genes, 94 were expressed in the tissues and conditions represented in Figure 4. The heatmap based on hierarchical clustering of expression levels revealed 10 putative NAC groups (A–J).

Transcript abundance of the teak NAC genes in group A (*Tg09g02930-XND1*, *Tg09g02550-VNI1,* and *Tg15g04300-VNI2)* was higher in secondary tissues. These TgNAC genes are homologous to Arabidopsis NAC genes involved in the SCW biosynthesis (Figure 4). In group F, TgNAC genes were preferentially transcribed in branches of 12-year-old trees. Only the genes *Tg01g12810-ANAC075* and *Tg02g05550-SND2* were homologous to the Arabidopsis NAC genes recognized to be involved in secondary tissue formation. As for group G, there are 13 NAC genes highly expressed in secondary tissues, from which only two (*Tg0519210-NST1* and *Tg02g15550-SND2)* are homologous to Arabidopsis genes involved in SCW formation. Most genes in the remaining groups were preferentially expressed in primary tissues (Figure 4). These data reveal several *T. grandis* NAC genes that seem to be involved in secondary growth and that will be better characterized and understood in further studies.

In Figure 4, the NAC genes marked in red were selected for qRT-PCR expression analysis due to their homology to Arabidopsis NAC genes involved in SCW formation. They are (*VND1-Tg09g04510; VND2-Tg11g04450*; *VND4-Tg15g08390*; *VND4-Tg15g11670*; *VND4-Tg16g07170*; *VND7-Tg03g10970*; *NST1-Tg05g19210*; *VNI2-Tg15g04300*; *XND1-Tg08g13700*; *SND2-Tg02g15550*; *ANAC075-Tg01g12810*; *ANAC33-Tg02g10510;* and *ANAC70-Tg03g13770*). Several of these genes showed high transcript abundance in teak primary tissues (roots, leaves, and flowers), suggesting function divergence from the split between both lineages.

### 3.6. Expression Analysis of Marker Genes for Secondary Cell Wall Formation at Different Tree Ages

**Figure 5:**
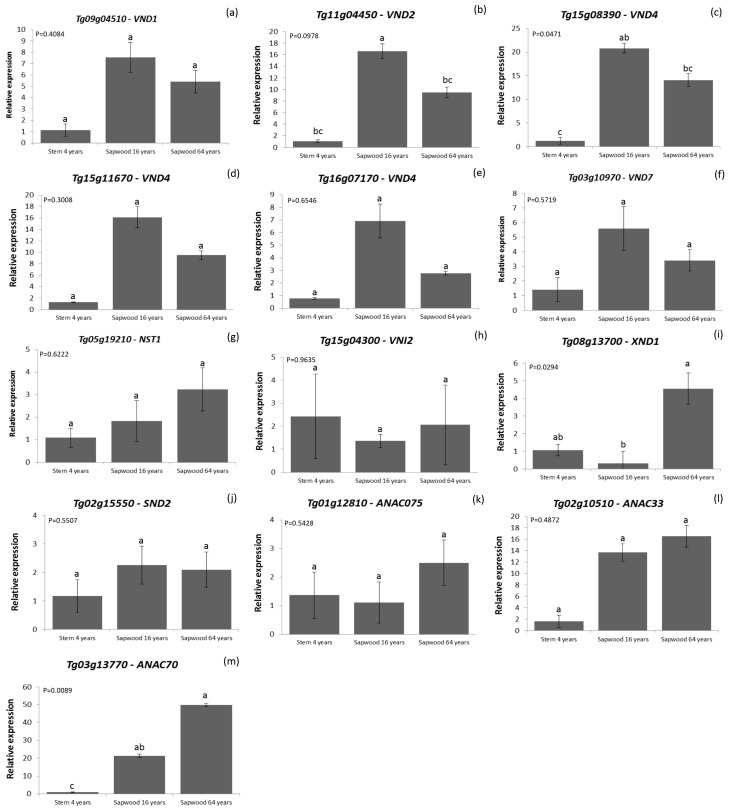
Expression levels of selected TgNAC genes using RT-qPCR. The relative expression of 13 selected NAC genes of *T. grandis* was normalized to the reference gene TgEF-1α in stem of 4-year-old trees and sapwood of 16- and 64-year-old trees. Bars represent standard errors (SEs) of three biological replicates and two technical replicates. The *p*-values are shown, and different letters represent statistically different means. Relative expression of the genes: (**a**) *VND1-Tg09g04510,* (**b**) *VND2-Tg11g04450,* (**c**) *VND4-Tg15g08390,* (**d**) *VND4-Tg15g11670,* (**e**) *VND4-Tg16g07170,* (**f**) *VND7-Tg03g10970,* (**g**) *NST1-Tg05g19210,* (**h**) *VNI2-Tg15g04300,* (**i**) *XND1-Tg08g13700,* (**j**) *SND2-Tg02g15550,* (**k**) *ANAC075-Tg01g12810,* (**l**) *ANAC33-Tg02g10510,* (**m**) *ANAC70-Tg03g13770*.

**Figure 6:**
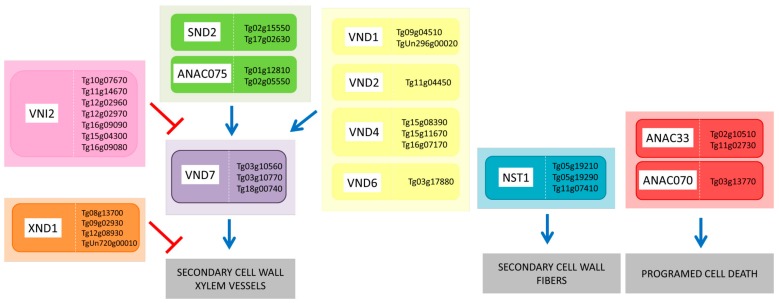
Schematic model of the transcriptional regulatory network controlling secondary cell wall biosynthesis in teak based in homologous genes of Arabidopsis. The NAC TFs VNDs (yellow and purple rectangles), SND2 (green rectangle), and ANAC075 (green rectangle) positively regulate SCW deposition in xylem vessels. TFs VNI2 (pink rectangle) and XND1 (orange rectangle) negatively regulate VND7 expression and differentiation of xylem vessels, respectively. NSTI (blue rectangle) is a transcriptional activator of SCW deposition in fibers, while ANAC33 and ANAC070 (red rectangles) are involved in programmed cell death. Blue and red arrows denote positive and negative regulation, respectively. Arabidopsis genes are represented in the white boxes and the putative homolog genes of teak are together in the same colored box.

The expression pattern of teak NAC genes that are homologous to Arabidopsis genes involved in SCW biosynthesis was analyzed to verify their functionality during teak wood formation in tissues of trees of different ages. Tissue samples were analyzed by quantitative real-time RT-PCR (RT-qPCR) in order to verify the expression of 13 selected TgNAC TF genes in stem from 4-year-old teak plants and in sapwood of 16- and 64-year-old teak trees (Figure 5). These 13 genes were selected based on their regulatory activity in the SCW formation pathway and programed cell death, both important events during wood formation (Figure 6) [68].

The process of wood formation comprises a series of consecutive events, starting with cell division in the vascular cambium and then followed by cell expansion and secondary cell wall deposition. The wood formation process ends in programmed cell death and heartwood formation [68]. Several transcription factors regulate the entire wood formation process, mainly the ones of the NAC and MYB families [10]. Among the NAC transcription factors regulating SCW deposition, VASCULAR-RELATED NAC-DOMAIN1-7 (VND1–VND7 proteins) are the main regulators of xylem vessel cell differentiation (Figure 6) [69]. In the present work, we analyzed the expression of four homologs of this family in *T. grandis* (*VND1-Tg09g04510*; *VND2-Tg11g04450*; *VND4-Tg15g08390*, *Tg15g11670*, *Tg16g07170*; and *VND7-Tg03g10970*). The relative expression mean for these VND genes followed a similar pattern. Of all VND genes analyzed, the highest expression was observed in 16-year-old trees, followed by the 64-year-old ones (Figure 5a–f). The 4-year-old plants presented the lowest expression among the age groups of trees analyzed. However, only the relative expression of the genes *VND2-Tg11g04450* and *VND4-Tg15g08390* showed statistically significant differences among the samples of teak trees (Figure 5b,c). These data may reflect the role of VND transcription factors during wood formation in *T. grandis* trees during diverse developmental stages, which are consistent with other analyzed species of trees. For instance, the SCW deposition in *Populus* developing wood is first seen in the vessel elements and in contact cells, and only occurs in fibers later on [70]. Likewise, in *Eucalyptus,* the wall thickness of fibers is increased and vessel frequencies and numbers decrease to supply the mechanical and physiological requirements during the tree development [71,72]. In *Pinus radiate,* cell wall-related transcripts are more abundant during its fast-growing phase, which occurs in the early-growth stages (9 years old), when compared to mature-growth (30 years old) [73]. The highest expression of VND genes was found in 16-year-old trees, which indicates the time of high secondary cell wall deposition in the ages analyzed.

Expression was also analyzed for the homolog gene of *NAC SECONDARY WALL THICKENING PROMOTING FACTOR 1* (*NST1-Tg05g19210*). These NST1 NAC TFs have been associated with secondary cell wall deposition in xylem fibers of Arabidopsis [24,31]. However, the analysis of the expression of *Tg05g19210* showed no statistically significant difference among the secondary tissues analyzed (Figure 5g). Therefore, it can be inferred that the *NST1–Tg05g19210* gene does not have a significative influence in SCW formation in teak.

In plant cells, the majority of NAC TFs are transcriptional activators, although transcriptional repressors are also present in the NAC gene family [74]. VND-INTERACTING2 (VNI2) is described in Arabidopsis as a transcriptional repressor that regulates differentiation of xylem cells by interacting with VND proteins and possibly with other NAC domain proteins [34]. We analyzed the expression of the *T. grandis Tg15g04300* gene, which is homologous to the *VNI2* in Arabidopsis (Figure 5h). There were no significant differences in the *Tg15g04300* expression among the teak trees of different ages. The VNI2 transcription factor represses the expression of genes regulated by VND7, which is a master regulator of xylem vessel differentiation [34]. In the analysis, also the expression of *VND7* homologue (*Tg03g10970*) was not altered during teak developmental stages, which indicates that perhaps VNI2 does not act as a repressor, or the repression may be occurring in all the teak stages analyzed.

Another repressor of secondary cell wall deposition is the XYLEM NAC DOMAIN1 (XND1) transcription factor (Figure 6). The gene of this TF is highly expressed in xylem and inhibits secondary cell wall deposition and autolysis in xylem vessels [75]. XND1 negatively regulates expression of genes involved in both programmed cell death and lignocellulose synthesis during secondary cell wall formation [29,76]. Interestingly, the expression of the teak repressor *Tg08g13700-XND1* (Figure 5i) was low in 4- and 16-year-old teak plants, but it was significantly higher in 64-year-old trees. These results corroborate the hypothesis that the secondary cell wall deposition is lower in older trees than in faster-growing younger trees. Apparently, the XND1 molecular mechanism of action is not related to VND TFs [77]. The NAC protein named SECONDARY WALL-ASSOCIATED NAC DOMAIN2 (SND2) is involved in the regulation of cellulose and hemicellulose biosynthesis (Figure 6). SND2 seems to occupy a subordinate place in the central layer of the transcriptional network of secondary cell wall formation [78]. There was no significant difference among the teak plants analyzed for the expression of the *Tg02g15550-SND2* gene (Figure 5j) among the teak plants analyzed, which could be acting in a secondary role in the secondary cell wall formation [78].

Another NAC transcription factor, the ARABIDOPSIS NAC DOMAIN CONTAINING PROTEIN 75 (ANAC075) is also involved in secondary cell wall biosynthesis and it is an upstream regulator of VND7 [75]. *ANAC075* overexpression induced ectopic secondary cell wall formation in Arabidopsis tissues through increasing expression of *VND7* [75,79]. Similarly, in *Populus*, higher expression of *PNAC127* (homologous of *ANAC075*) was observed in differentiating xylem [6]. No difference in the expression of the teak *ANAC075* homolog *Tg01g12810* (Figure 5k) was observed in our analysis. Probably, the other NAC transcription factors have a stronger influence than *Tg01g12810* during the SCW formation in the *T. grandis* tissues analyzed.

We also analyzed TFs ANAC033 and ANAC070 that are known to regulate programmed cell death, the last step of secondary growth. The highest expression of the *ANAC70* teak homologue (*Tg03g13770)* was observed in 16- and 64-year-old trees (Figure 5m). This result indicates that this gene might have a key function in programed cell death of *T. grandis* secondary tissues. While, the evaluation of *ANAC33 (Tg02g10510)* homologue expression, showed no statistically significant difference between the three ages analyzed (Figure 5l). Apparently, these two Arabidopsis homologous proteins have a more prominent role in regulating lateral root cap formation than in regulation of secondary cell wall deposition [80].

## 4. Conclusions

Here we presented a genome-wide analysis of *T. grandis* NAC domain genes with identification and characterization of NAC transcriptional factors homologous to Arabidopsis and that are related to SCW formation. Analysis of teak NAC TFs encoding genes identified 130 NAC proteins, from which 116 represented non-redundant proteins coded by 107 loci. These identified 116 *T. grandis* NAC proteins were phylogenetically clustered into 23 distinct clades in evolutionary relationships with Arabidopsis, *Populus,* and *Eucalyptus* trees. Many of the TgNAC genes were located adjacent to each other, indicating that they might have resulted from duplication events. Most genes in groups A and F were preferentially expressed in secondary tissues, where they presented higher transcript abundance. As for the remaining groups, most NAC genes were highly expressed in flowers, roots, leaves, and seedlings. Considering the regulation function of the NAC genes in SCW biosynthesis, 13 NAC genes were analyzed for their expression in various tissues of three developmental stages of teak trees. Data from RT-qPCR revealed highest expression of VND genes *VND2-Tg11g04450* and *VND4-Tg15g08390* in tissues of 16-year-old trees. VND TFs are related to xylem vessels formation, indicating that these two VND genes (*Tg11g04450* and *Tg15g08390*) possibly participate in the regulation of *T. grandis* SCW deposition. In addition, the NAC homologous repressor *XND1* (*Tg08g13700*) presented highest expression in 64-year-old tissues and gave support to the hypothesis of lower secondary cell wall deposition in older trees. Data reported in the present work contribute to the understanding of structure and functionality of NAC transcription factor genes in *T. grandis* and they are potentially useful for future studies aiming to improve biomass and wood quality using biotechnological approaches.

## Supplementary Materials

The following tables and figures are available online at https://www.mdpi.com/2073-4425/11/1/20/s1: Table S1. Primers used for gene expression analysis by RT-qPCR; Table S2. Teak NAC gene family; Table S3. Teak NAC proteins motifs; Table S4. Cellular localization of *Tectona grandis* NAC proteins; Table S5. Genomic location of NACs in the genome of *Tectona grandis*; Table S6. Transcript abundances of *Tectona grandis* NAC genes, which were estimated using cufflinks RNAseq experiment atlas from NCBI SRA BioProject PRJNA287604.; Figure S1. Phylogenetic profile of 28 plant species and numbers of identified NAC proteins; Figure S2. Duplication events in Arabidopsis, *Eucalyptus*, *Populus,* and *T. grandis*.
