# Supplementary material for: Analysis of NAC Domain Transcription Factor Genes of Tectona grandis L.f. Involved in Secondary Cell Wall Deposition
Source: Genes (Basel). 2019 Dec 23;11(1):20. doi: 10.3390/genes11010020 (PMC7016782; doi:10.3390/genes11010020)
Supplement: Supplementary file 1 [file genes-11-00020-s001.zip › Reviewed_round 2 _Supplementary material_Analysis of NAC Domain Transcription Factor Genes involved in Secondary Cell Wall deposition in Tectona grandis.pdf]

Supplementary Materials

Analysis of NAC Domain Transcription Factor Genes of *Tectona grandis* Involved in Secondary Cell Wall Deposition

Fernando Manuel Matias Hurtado[1,3], Maísa de Siqueira Pinto[1,4], Perla Novais de Oliveira[1,5], Diego Mauricio Riaño-Pachon[2,6],Laura Beatriz Inocente[1,7] and Helaine Carrer [1,8]*

[1]Department of Biological Sciences, Luiz de Queiroz College of Agriculture (ESALQ), University of São Paulo, Av. Pádua Dias, 11, CP 9, Piracicaba, SP 13418-900, SP, Brazil. [3]fermathur1992@usp.br; [4]maisadesiqueira@gmail.com; [5]perla.oliveira@usp.br; [7]laurabinocente@gmail.com; [8]hecarrer@usp.br

[2] Computational, Evolutionary and Systems Biology Laboratory, Center of Nuclear Energy in Agriculture, CENA, University of São Paulo. Av. Centenário 303, Piracicaba SP 13416-000, SP, Brazil. [6]diego.riano@cena.usp.br

* Corresponding author: hecarrer@usp.br; Tel.: +55 19 3429.4344, Ext. 21.

FMMH and MSP contributed equally to the manuscript

Table S1. Primers used for gene expression analysis by RT-qPCR.

Gene	Reference Number	Primers
ANAC075[a]	Tg01g12810	FW - 5` TCCACGTCCCAAGAACCATG 3`<br>RV - 5` GCAGCCCATGATGACTTCCT 3`
ANAC33[b]	Tg02g10510	FW - 5` ACGGGCAGAAGACTGATTGG 3`<br>RV - 5` TGGAAAGGTACAACGGGCTC 3`
SND2[c]	Tg02g15550	FW - 5` CATCTCCGCTGATCCCGAAT 3`<br>RV - 5` ATTCCTCGTCGCTGCTAACC 3`
VND7[d]	Tg03g10970	FW - 5` CCCACAATGATGACTTAGATGCTC 3`<br>RV - 5` CGTTCCAACTTGGAGAAGGA 3`
ANAC70[e]	Tg03g13770	FW - 5` TGAGAGTGCAAGTCCAGCAG 3`<br>RV - 5` ACAAGGCGATCCCATTCGTT 3`
NST1[f]	Tg05g19210	FW - 5` CCACAGGGACACGCACTAAT 3`<br>RV - 5` GGGGCACGACCTTTGTAGAA 3`
XND1[g]	Tg08g13700	FW - 5` GCCATCCAGATGTCATCCCA 3`<br>RV - 5` TCGACCCCTATAGTCTGCCA 3`
VND1[h]	Tg09g04510	FW - 5` ATCCCACAACTTGGTGTGCA 3`<br>RV - 5` CCTTTTCGTTGGTGGCATGG 3`
VND2[i]	Tg11g04450	FW - 5` CGTTCAATCTATGATGCCACTGG 3`<br>RV - 5` AAGTGTACCTGTTCCTCCCC 3`
VNI2[j]	Tg15g04300	FW - 5` CCCCTTGCCAGCTTCCATAA 3`<br>RV - 5` TGTTGCCTTCCAGTAGCCAG 3`
VND4[k]	Tg15g11670	FW - 5` TTCTTCCACAATTCCGACGG 3`<br>RV - 5` GCCAAACAGTCCTCTTCCAT 3`
VND4[l]	Tg15g08390	FW - 5` GCAATTTGCAGTCATCAGGA 3`<br>RV - 5` GGGATGCCACAAACTTGTCT 3`
VND4[m]	Tg16g07170	FW - 5` CGAGCCCCAAATGGACAGAA 3`<br>RV - 5` TCCTTGTGGTTGCCATCCTC 3`

[a]*ARABIDOPSIS NAC-DOMAIN CONTAINING PROTEIN 75*, [b]*ARABIDOPSIS NAC-DOMAIN CONTAINING PROTEIN 33*, [c]*SECUNDARY WALL-ASSOCIATED NAC DOMAIN 2*, [d]*VASCULAR-RELATED NAC-DOMAIN 7*, [e]*ARABIDOPSIS NAC-DOMAIN CONTAINING PROTEIN 70*, [f]*NAC SECUNDARY WALL THICKENING PROMOTING FACTOR*, [g]*XYLEM NAC DOMAIN 1*, [h]*VASCULAR-RELATED NAC-DOMAIN 1*, [i]*VASCULAR-RELATED NAC-DOMAIN 2*, [j]*VDN-INTERACTING 2*, [k]*VASCULAR-RELATED NAC-DOMAIN 4*, [l]*VASCULAR-RELATED NAC-DOMAIN 4*, [m]*VASCULAR-RELATED NAC-DOMAIN 4.*

Table S2. Teak NAC gene family.

Gene locus	Gene Code	Arabidopsis locus description	Arabidopsis ortholog locus	Score	E-value
Tg01g00150	TGNAC001	ANAC071	AT4G17980.1	288	2E-32
Tg01g01920	TGNAC002	ANAC071	AT4G17980.1	301	2E-34
Tg01g08840	TGNAC003	ANAC087	AT5G18270.1	702	7E-91
Tg01g08850	TGNAC004	ANAC087	AT5G18270.1	704	1E-91
Tg01g11480	TGNAC005	ANA072, RD26	AT4G27410.2	460	6E-55
Tg01g12810.t1	TGNAC006	ANAC075	AT4G29230.1	1205	1E-164
Tg01g12810.t3	TGNAC007	ANAC075	AT4G29230.1	1181	1E-160
Tg01g17760	TGNAC008	ANAC090	AT5G22380.1	606	3E-80
Tg02g02730	TGNAC009	ANAC087	AT5G18270.1	746	4E-98
Tg02g05550	TGNAC010	ANAC075	AT4G29230.1	1309	1E-179
Tg02g09900	TGNAC011	ANAC042	AT2G43000.1	686	7E-91
Tg02g09660	TGNAC012	ANAC021	AT1G56010.2	762	1E-101
Tg02g10410	TGNAC013	ANAC020	AT1G54330.1	750	1E-100
Tg02g10510	TGNAC014	ANAC033	AT1G79580.1	706	2E-92
Tg02g14690	TGNAC015	ANAC025	AT1G61110.1	729	8E-97
Tg02g15550	TGNAC016	ANAC073, SND2	AT4G28500.1	890	1E-121
Tg02g15570	TGNAC017	ANAC029, NAC-like, activated by AP3/PI	AT1G69490.1	309	2E-36
Tg03g08530	TGNAC018	ANAC002, ATAF1	AT1G01720.1	933	1E-128
Tg03g10560	TGNAC019	ANAC030, VND7	AT1G71930.1	726	4E-96
Tg03g10970	TGNAC020	ANAC030, VND7	AT1G71930.1	726	4E-96
Tg03g13770	TGNAC021	ANAC070	AT4G10350.1	807	1E-107
Tg03g18100	TGNAC022	ANAC020	AT1G54330.1	199	6E-20
Tg03g17880	TGNAC023	ANAC101, VND6	AT5G62380.1	207	9E-20
Tg03g17890	TGNAC024	ANAC032	AT1G77450.1	161	2E-14
Tg03g18120	TGNAC025	ANAC102	AT5G63790.1	199	7E-19
Tg05g04990	TGNAC026	ANAC038	AT2G24430.2	772	1E-103
Tg05g10730	TGNAC027	ANAC074	AT4G28530.1	685	6E-90
Tg05g12200	TGNAC028	ANAC035	AT2G02450.2	855	1E-113
Tg05g19210	TGNAC029	ANAC043, NSTI	AT2G46770.1	896	1E-120
Tg05g19290	TGNAC030	ANAC043, NSTI	AT2G46770.1	889	1E-119
Tg06g07710	TGNAC031	ANAC002, ATAF1	AT1G01720.1	432	1E-50
Tg06g07670	TGNAC032	ANAC072, RD26	AT4G27410.2	432	1E-52
Tg06g07740	TGNAC033	ANAC072	AT4G27410.2	228	4E-23
Tg06g07750	TGNAC034	ANAC019	AT1G52890.1	216	9E-22
Tg06g11580	TGNAC035	ANAC035	AT2G02450.2	849	1E-112
Tg06g16210	TGNAC036	ANAC041	AT2G33480.1	407	7E-50
Tg06g17630	TGNAC037	ANAC014	AT5G24590.2	699	1E-86
Tg07g01830	TGNAC038	ANAC052	AT3G10490.2	742	1E-95
Tg07g01840	TGNAC039	ANAC053	AT3G10500.1	913	1E-117
Tg07g09430	TGNAC040	ANAC002, ATAF1	AT1G01720.1	691	2E-92
Tg07g12160	TGNAC041	ANAC028	AT1G65910.1	1187	1E-157
Tg08g06610.t1	TGNAC042	ANAC017	AT1G34190.1	1059	1E-139

Tg08g06610.t2	TGNAC043	ANAC017	AT1G34190.1	332.8	5.6E-107
Tg08g06620	TGNAC044	ANAC017	AT1G34190.1	1077	1E-142
Tg08g07120	TGNAC045	ANAC003	AT1G76420.1	768	1E-101
Tg08g13700	TGNAC046	ANAC104, XND1	AT5G64530.1	518	1E-68
Tg08g17690	TGNAC047	ANAC036	AT2G17040.1	680	5E-91
Tg09g04510	TGNAC048	ANAC037, VND1	AT2G18060.1	1072	1E-147
Tg09g02550.t1	TGNAC049	ANAC082, VNI1	AT5G09330.3	692	1E-85
Tg09g02550.t2	TGNAC050	ANAC082, VNI1	AT5G09330.3	692	4E-87
Tg09g02550.t6	TGNAC051	ANAC082, VNI1	AT5G09330.3	693	5E-87
Tg09g02930	TGNAC052	ANAC104, XND1	AT5G64530.1	426	2E-54
Tg09g03360	TGNAC053	ANAC002, ATAF1	AT1G01720.1	908	1E-124
Tg09g15630	TGNAC054	ANAC009	AT1G26870.1	785	1E-102
Tg09g15920	TGNAC055	ANAC100	AT5G61430.1	879	1E-118
Tg10g01380	TGNAC056	ANAC057	AT3G17730.1	847	1E-117
Tg10g05660	TGNAC057	ANAC100	AT5G61430.1	930	1E-126
Tg10g05820	TGNAC058	ANAC060	AT3G44290.1	162	7E-14
Tg10g06080	TGNAC059	ANAC032	AT1G77450.1	127	2E-09
Tg10g07670	TGNAC060	ANAC083, VNI2	AT5G13180.1	456	1E-57
Tg11g07410	TGNAC061	ANAC043 NSTI	AT2G46770.1	808	1E-107
Tg11g02730	TGNAC062	ANAC033	AT1G79580.1	98	0,000007
Tg11g02740	TGNAC063	ANAC052	AT3G10490.2	86	0,00001
Tg11g03710	TGNAC064	ANAC100	AT5G61430.1	939	1E-128
Tg11g04450	TGNAC065	ANAC076, VND2	AT4G36160.1	166	1E-14
Tg11g05530	TGNAC066	ANAC058	AT3G18400.1	775	1E-103
Tg11g07580	TGNAC067	NAC TRANSCRIPTION FACTOR-LIKE 9, NTL9	AT4G35580.2	652	9E-85
Tg11g10750	TGNAC068	ANAC008	AT1G25580.1	1237	1E-170
Tg11g12110	TGNAC069	ANAC009	AT1G26870.1	805	1E-105
Tg11g11960	TGNAC070	ANAC029, NAC-like, activated by AP3/PI	AT1G69490.1	789	1E-107
Tg11g14670	TGNAC071	ANAC083, VNI2	AT5G13180.1	593	3E-78
Tg12g02960	TGNAC072	ANAC083, VNI2	AT5G13180.1	169	4E-15
Tg12g02970	TGNAC073	ANAC083, VNI2	AT3G10480.2	184	2E-16
Tg12g03010	TGNAC074	ANAC050	AT3G10480.2	747	5E-96
Tg12g03030	TGNAC075	ANAC053	AT3G10500.1	1027	1E-135
Tg12g08930.t1	TGNAC076	ANAC104, XND1	AT5G64530.1	486	9E-64
Tg12g08930.t3	TGNAC077	ANAC104, XND1	AT5G64530.1	319	6E-39
Tg12g10590	TGNAC078	ANAC054/CUC1	AT3G15170.1	106	0,0000007
Tg12g17130.t1	TGNAC079	ANAC036	AT2G17040.1	773	1E-104
Tg12g17130.t2	TGNAC080	ANAC036	AT2G17040.1	582	9E-76
Tg13g00820.t1	TGNAC081	ANAC042	AT2G43000.1	649	1E-85
Tg13g00820.t2	TGNAC082	ANAC042	AT2G43000.1	433	3E-54
Tg13g06720	TGNAC083	ANAC098, CUC2	AT5G53950.1	808	1E-107
Tg13g07150.t1	TGNAC084	ANAC072, RD26	AT4G27410.2	959	1E-131
Tg13g07150.t2	TGNAC085	ANAC072, RD26	AT4G27410.2	909	1E-123
Tg13g09540	TGNAC086	ANAC090	AT5G22380.1	568	2E-74
Tg13g09550	TGNAC087	ANAC090	AT5G22380.1	576	1E-75

Tg13g12990	TGNAC088	ANAC081	AT5G08790.1	346	2E-41
Tg14g02070	TGNAC089	ANAC040	AT2G27300.1	595	6E-75
Tg14g02200	TGNAC090	ANAC090	AT5G22380.1	647	3E-87
Tg14g03970	TGNAC091	ANAC056	AT3G15510.1	324	7E-36
Tg14g11050.t1	TGNAC092	ANAC074	AT4G28530.1	711	2E-94
Tg14g11050.t2	TGNAC093	ANAC074	AT4G28530.1	714	2E-94
Tg15g01040	TGNAC094	ANAC008	AT1G25580.1	719	1E-91
Tg15g02070	TGNAC095	ANAC0100	AT5G61430.1	878	1E-118
Tg15g04300	TGNAC096	ANAC083 VNI2	AT5G13180.1	744	1E-101
Tg15g08390	TGNAC097	ANAC007, VND4	AT1G12260.1	211	5E-21
Tg15g10730	TGNAC098	ANAC009	AT1G26870.1	759	1E-98
Tg15g11030	TGNAC099	ANAC029, NAC-like, activated by AP3/PI	AT1G69490.1	830	1E-113
Tg15g11670	TGNAC100	ANAC007, VND4	AT1G12260.1	979	1E-133
Tg16g02600	TGNAC101	ANAC039	AT2G24430.2	640	3E-83
Tg16g07170	TGNAC102	ANAC007, VND4	AT1G12260.1	979	1E-133
Tg16g09080	TGNAC103	ANAC083, VNI2	AT5G13180.1	422	1E-52
Tg16g09090	TGNAC104	ANAC083, VNI2	AT5G13180.1	447	2E-56
Tg16g13420	TGNAC105	ANAC057	AT3G17730.1	589	6E-78
Tg17g02630	TGNAC106	ANAC073, SND2	AT4G28500.1	871	1E-118
Tg17g08390	TGNAC107	ANAC072, RD26	AT4G27410.2	899	1E-122
Tg17g09060	TGNAC108	ANAC098, CUC2	AT5G53950.1	697	4E-90
Tg17g08380	TGNAC109	ANAC056	AT3G15510.1	891	1E-120
Tg18g00740	TGNAC110	ANAC030, VND7	AT1G71930.1	780	1E-104
Tg18g04440	TGNAC111	ANAC041	AT2G33480.1	149	6E-12
Tg18g09420	TGNAC112	ANAC032	AT1G77450.1	503	1E-58
Tg18g11300.t1	TGNAC113	ANAC090	AT5G22380.1	573	3E-75
Tg18g11300.t2	TGNAC114	ANAC090	AT5G22380.1	425	1E-53
TgUn272g00030	TGNAC115	ANAC002, ATAF1	AT1G01720.1	939	1E-129
TgUn296g00020	TGNAC116	ANAC037, VND1	AT2G18060.1	253	3E-28
TgUn720g00010	TGNAC117	ANAC104, XND1	AT5G64530.1	366	2E-46

Table S3. Teak NAC proteins motifs. Logos of 10 motifs identified in TgNAC proteins using MEME, the E-value, motif consensus, and name annotation based previously published work (* *Eucaliptus* [5] motif consenso and ** *Populus* [6] motif consenso) are presented.

Motif	E – value	Motif consenso	Name anotation
Motif 1 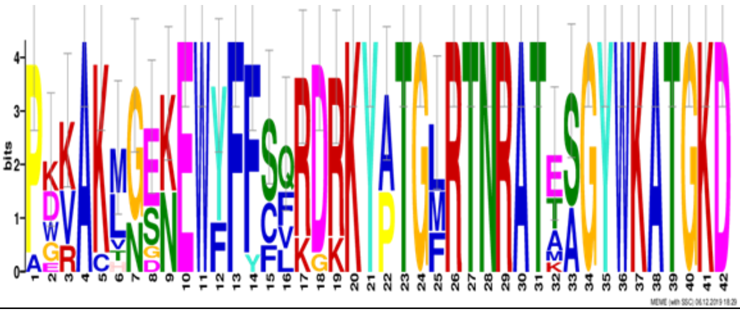	1.90e-136	motif3*	NAC subdomain C
		motif4*	NAC subdomain D
Motif 2 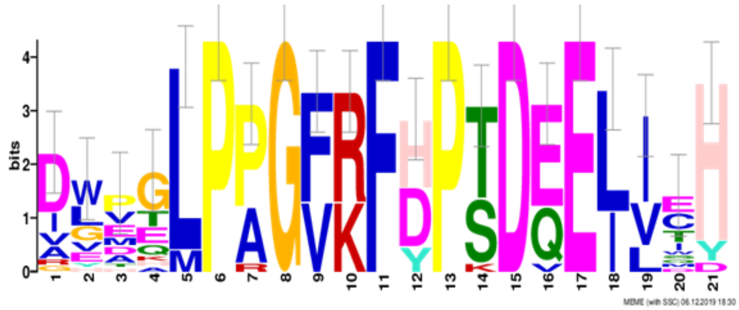	3.93e-146	motif1*	NAC subdomain A
Motif 3 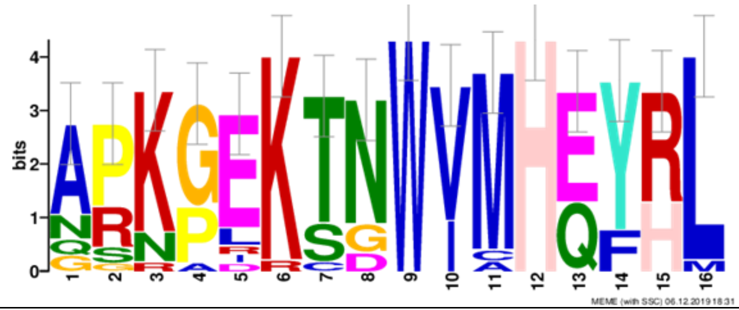	2.21e-173	motif6*	NAC subdomain D
Motif 4 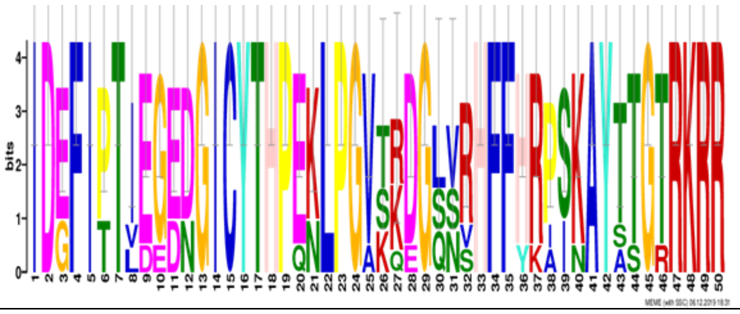	1.01e-281	Motif9*	–
Motif 5 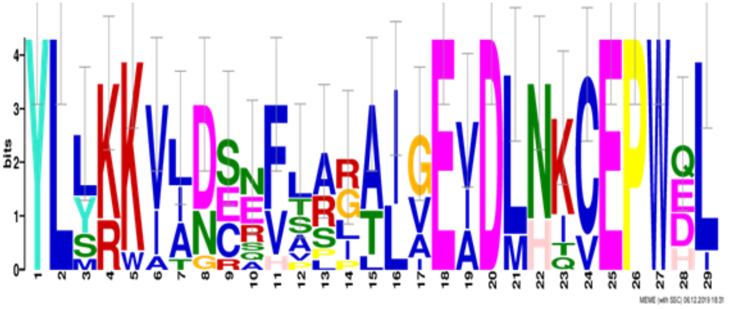	5.16e-281	motif2*	NAC subdomain B

Motif 6 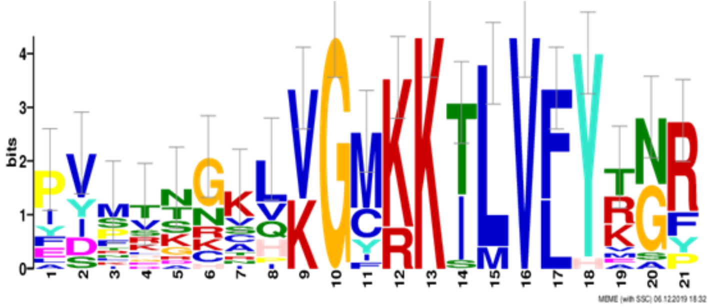	1.09e-172	motif5*	NAC subdomain D
Motif 7 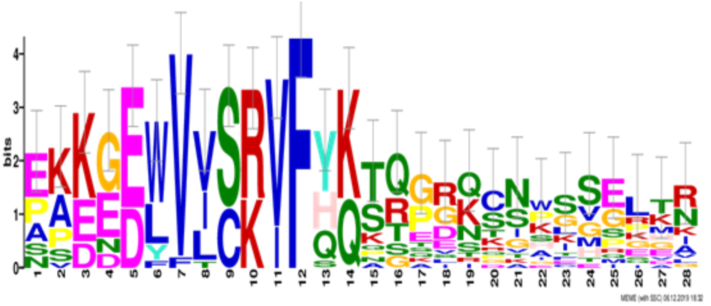	7.51e-177	motif7*	NAC subdomain E
Motif 8 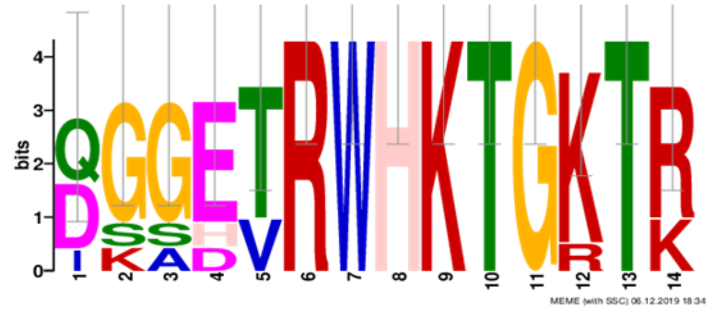	1.22e-46	motif10**	–
Motif 9 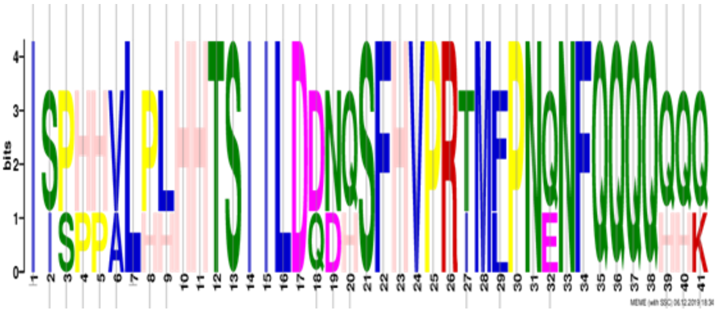	2.91e-206	–	–
Motif 10 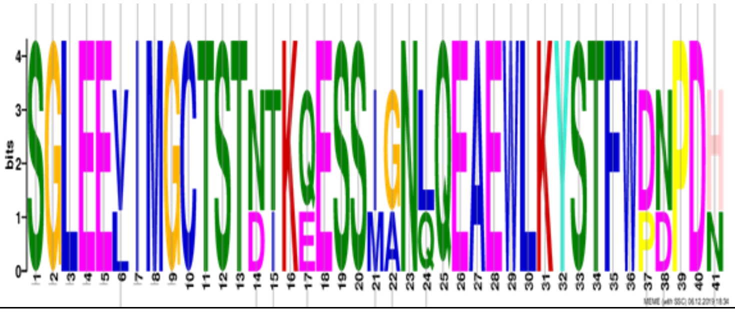	7.82e-284	–	–

Table S4. Cellular localization of *Tectona grandis* NAC proteins.

Protein ID	Localization BUSCA	Localization CELLO
Tg01g00150	nucleus	nucleus
Tg01g01920	nucleus	nucleus
Tg01g08840	nucleus	nucleus
Tg01g08850	nucleus	nucleus
Tg01g11480	nucleus	nucleus
Tg01g12810	nucleus	nucleus
Tg01g17760	nucleus	nucleus
Tg02g02730	nucleus	nucleus
Tg02g05550	nucleus	nucleus
Tg02g09660	nucleus	nucleus
Tg02g09900	nucleus	nucleus
Tg02g10410	nucleus	nucleus
Tg02g10510	nucleus	cytoplasm
Tg02g14690	nucleus	nucleus
Tg02g15550	nucleus	nucleus
Tg02g15570	chloroplast	nucleus
Tg03g08530	nucleus	nucleus
Tg03g10560	nucleus	nucleus
Tg03g10970	nucleus	nucleus
Tg03g13770	nucleus	nucleus
Tg03g17880	nucleus	cytoplasm
Tg03g17890	nucleus	plasma membrane
Tg03g18100	chloroplast	chloroplast
Tg03g18120	nucleus	cytoplasm
Tg05g04990	nucleus	nucleus
Tg05g10730	nucleus	nucleus
Tg05g12200	nucleus	nucleus
Tg05g19210	nucleus	nucleus
Tg05g19290	nucleus	nucleus
Tg06g07670	nucleus	nucleus
Tg06g07710	nucleus	nucleus
Tg06g07740	nucleus	nucleus
Tg06g07750	nucleus	nucleus
Tg06g11580	nucleus	nucleus
Tg06g16210	nucleus	nucleus
Tg06g17630	endomembrane system	nucleus
Tg07g01830	nucleus	nucleus
Tg07g01840	endomembrane system	nucleus
Tg07g09430	nucleus	nucleus
Tg07g12160	endomembrane system	nucleus
Tg08g06610	endomembrane system	nucleus
Tg08g06620	endomembrane system	nucleus
Tg08g07120	nucleus	nucleus
Tg08g13700	nucleus	nucleus
Tg08g17690	nucleus	nucleus

Tg09g02550	nucleus	nucleus
Tg09g02930	nucleus	cytoplasm
Tg09g03360	nucleus	nucleus
Tg09g04510	nucleus	nucleus
Tg09g15630	nucleus	nucleus
Tg09g15920	nucleus	nucleus
Tg10g01380	nucleus	nucleus
Tg10g05660	nucleus	nucleus
Tg10g05820	nucleus	nucleus
Tg10g06080	nucleus	extracellular space
Tg10g07670	nucleus	nucleus
Tg11g02730	nucleus	cytoplasm
Tg11g02740	extracellular space	cytoplasm
Tg11g03710	nucleus	cytoplasm
Tg11g04450	nucleus	cytoplasm
Tg11g05530	nucleus	nucleus
Tg11g07410	nucleus	nucleus
Tg11g07580	nucleus	cytoplasm
Tg11g10750	nucleus	nucleus
Tg11g11960	nucleus	nucleus
Tg11g12110	nucleus	nucleus
Tg11g14670	nucleus	nucleus
Tg12g02960	nucleus	nucleus
Tg12g02970	nucleus	nucleus
Tg12g03010	nucleus	nucleus
Tg12g03030	endomembrane system	nucleus
Tg12g08930	nucleus	nucleus
Tg12g10590	nucleus	nucleus
Tg12g17130	nucleus	cytoplasm
Tg13g00820	nucleus	nucleus
Tg13g06720	nucleus	nucleus
Tg13g07150	nucleus	nucleus
Tg13g09540	nucleus	nucleus
Tg13g09550	nucleus	nucleus
Tg13g12990	nucleus	nucleus
Tg14g02070	plasma membrane	nucleus
Tg14g02200	nucleus	cytoplasm
Tg14g03970	nucleus	nucleus
Tg14g11050	nucleus	cytoplasm
Tg15g01040	nucleus	nucleus
Tg15g02070	nucleus	nucleus
Tg15g04300	nucleus	nucleus
Tg15g08390	nucleus	nucleus
Tg15g10730	nucleus	nucleus
Tg15g11030	nucleus	nucleus
Tg15g11670	nucleus	nucleus
Tg16g02600	nucleus	nucleus
Tg16g07170	nucleus	nucleus

Tg16g09080	nucleus	nucleus
Tg16g09090	nucleus	nucleus
Tg16g13420	nucleus	nucleus
Tg17g02630	nucleus	nucleus
Tg17g08380	nucleus	nucleus
Tg17g08390	nucleus	nucleus
Tg17g09060	nucleus	plasma membrane
Tg18g00740	nucleus	nucleus
Tg18g04440	nucleus	nucleus
Tg18g09420	extracellular space	nucleus
Tg18g11300	nucleus	nucleus
TgUn296g00020	chloroplast	cytoplasm
TgUn272g00030	nucleus	nucleus
TgUn720g00010	nucleus	nucleus

Table S5. Genomic location of NACs in the genome of *Tectona grandis*.

Gene_ID	Pseudomolecule	Gene Start (pb)	Gene End (pb)
Tg01g00150	01	100651	102109
Tg01g01920	01	1272763	1274227
Tg01g08840	01	6650521	6652802
Tg01g08850	01	6655440	6657402
Tg01g11480	01	8960430	8962139
Tg01g12810	01	10560086	10565923
Tg01g17760	01	17473607	17475180
Tg02g02730	02	2368292	2370614
Tg02g05550	02	5861368	5866533
Tg02g09660	02	11444805	11448869
Tg02g09900	02	11642247	11644252
Tg02g10410	02	12125444	12127643
Tg02g10510	02	12212042	12216373
Tg02g14690	02	15067485	15068720
Tg02g15550	02	15691858	15695487
Tg02g15570	02	15707390	15708003
Tg03g08530	03	10338456	10340163
Tg03g10560	03	11813720	11815838
Tg03g10970	03	12085352	12087581
Tg03g13770	03	13974114	13977147
Tg03g17880	03	17073920	17075388
Tg03g17890	03	17083750	17089834

Tg03g18100	03	17311080	17312660
Tg03g18120	03	17324513	17325847
Tg05g04990	05	3563051	3567504
Tg05g10730	05	8509349	8512085
Tg05g12200	05	11165686	11168157
Tg05g19210	05	17430830	17432430
Tg05g19290	05	17510868	17512817
Tg06g07670	06	5426077	5427523
Tg06g07710	06	5461617	5463672
Tg06g07740	06	5481308	5482406
Tg06g07750	06	5484991	5486584
Tg06g11580	06	9451036	9453886
Tg06g16210	06	15389293	15391097
Tg06g17630	06	16308386	16311887
Tg07g01830	07	1417889	1422065
Tg07g01840	07	1426175	1430978
Tg07g09430	07	9986626	9988534
Tg07g12160	07	13653345	13657814
Tg08g06610	08	4671777	4675732
Tg08g06620	08	4676644	4679788
Tg08g07120	08	5045264	5048014
Tg08g13700	08	13606310	13607704
Tg08g17690	08	16640344	16641591
Tg09g02550	09	2108466	2115424
Tg09g02930	09	2532679	2534247
Tg09g03360	09	3008524	3010544
Tg09g04510	09	4929000	4931759
Tg09g15630	09	15581846	15583632
Tg09g15920	09	15875987	15877920
Tg10g01380	10	825541	828407
Tg10g05660	10	4133932	4135739
Tg10g05820	10	4296708	4297518
Tg10g06080	10	4539325	4547787
Tg10g07670	10	6369428	6370530
Tg11g02730	11	2364113	2364779
Tg11g02740	11	2369832	2370644

Tg11g03710	11	3287731	3289306
Tg11g04450	11	4346122	4347447
Tg11g05530	11	6258532	6261052
Tg11g07410	11	9693204	9695369
Tg11g07580	11	9919320	9922886
Tg11g10750	11	13053622	13058541
Tg11g11960	11	13937913	13939282
Tg11g12110	11	14064504	14066034
Tg11g14670	11	15902286	15903495
Tg12g02960	12	2124414	2125307
Tg12g02970	12	2127026	2127923
Tg12g03010	12	2159430	2165896
Tg12g03030	12	2169965	2174339
Tg12g08930	12	9005829	9007473
Tg12g10590	12	10658980	10659737
Tg12g17130	12	15364039	15365527
Tg13g00820	13	506214	507523
Tg13g06720	13	6415767	6418101
Tg13g07150	13	7167495	7169625
Tg13g09540	13	10758767	10760459
Tg13g09550	13	10784952	10787577
Tg13g12990	13	13515197	13517742
Tg14g02070	14	1278566	1284409
Tg14g02200	14	1361124	1362421
Tg14g03970	14	2885920	2887672
Tg14g11050	14	10646580	10650310
Tg15g01040	15	920180	923969
Tg15g02070	15	2810419	2812071
Tg15g04300	15	6384214	6385961
Tg15g08390	15	9676357	9680089
Tg15g10730	15	11502116	11503715
Tg15g11030	15	11702280	11703828
Tg15g11670	15	12098199	12100357
Tg16g02600	16	1973134	1976925
Tg16g07170	16	6673219	6677803
Tg16g09080	16	10385023	10386605

Tg16g09090	16	10395308	10397373
Tg16g13420	16	14015643	14018968
Tg17g02630	17	2124067	2126017
Tg17g08380	17	8935438	8937265
Tg17g08390	17	8955890	8957571
Tg17g09060	17	9535173	9536792
Tg18g00740	18	434746	436426
Tg18g04440	18	3341645	3345250
Tg18g09420	18	6931431	6933095
Tg18g11300	18	8596625	8598152
TgUn296g00020	Scaffold_Un296	36115	36486
TgUn272g00030	Scaffold_Un272	23446	25033
TgUn720g00010	Scaffold_Un720	19039	19583

Table S6. Transcript abundances of *Tectona grandis* NAC genes.

Gene_ID	Seedlings[a]	Roots[b]	Leaves[c]	Flowers[d]	Stem 12yr.1[e]	Stem 12yr.2[f]	Stem 60yr.1[g]	Stem 60yr.2[h]	Branch 12yr.1[i]	Branch 12yr.2[j]	Branch 60yr.1[k]	Branch 60yr.2[l]
Tg09g02930	1.14	14.62	3.00	2.10	2.88	3.24	26.13	8.98	26.30	40.57	56.25	60.54
Tg09g02550	15.68	37.60	50.74	44.27	39.15	30.50	44.52	62.54	96.97	48.77	73.94	63.96
Tg15g04300	27.19	89.45	21.17	74.12	115.99	107.31	133.21	195.30	273.85	165.66	171.05	162.73
Tg16g02600	0.30	1.28	0.19	1.05	1.02	3.26	1.30	1.39	0.65	3.18	1.32	0.12
Tg06g07670	0.00	0.00	0.00	0.16	0.00	0.44	0.00	0.00	0.00	0.00	0.00	0.00
Tg06g11580	5.54	5.59	13.40	2.02	16.98	11.45	11.15	11.72	5.17	6.42	9.33	10.22
Tg14g02070	6.91	6.08	17.59	12.89	16.65	17.35	19.33	26.57	13.52	13.53	29.66	23.38
Tg13g00820	4.45	3.00	0.84	0.72	1.74	1.98	0.67	0.00	2.82	0.85	1.85	2.14
Tg13g09550	0.52	0.51	0.00	0.20	0.37	0.30	0.00	0.00	0.39	0.00	0.00	0.21
Tg02g14690	28.51	4.63	27.27	18.34	0.00	0.21	0.76	1.87	16.73	0.97	1.58	2.13
Tg01g17760	1.71	0.48	0.75	0.00	0.00	0.00	0.00	0.00	0.00	0.00	0.00	0.00
Tg01g08840	0.59	0.22	0.00	0.13	0.00	0.00	0.00	0.00	0.00	0.00	0.00	0.00
Tg02g15570	0.25	0.00	0.00	0.00	0.00	0.00	0.00	0.00	0.00	0.00	0.00	0.00
Tg15g10730	0.19	1.78	0.60	0.14	0.88	0.18	0.20	1.10	1.62	0.19	0.99	0.69
Tg03g10970	1.00	3.73	3.28	0.66	0.00	0.00	0.00	0.00	0.51	0.00	0.00	0.00
Tg03g13770	0.35	2.96	1.75	0.00	0.00	0.00	0.00	0.00	0.00	0.00	0.00	0.00

Tg12g17130	1.18	21.98	4.66	1.61	1.39	1.58	0.54	0.20	2.71	0.34	1.61	0.73
Tg05g04990	1.34	19.87	0.23	4.50	0.00	0.38	0.00	0.00	0.00	0.00	0.00	0.09
Tg08g13700	1.91	38.65	0.16	4.20	0.00	0.00	0.00	0.00	0.81	1.58	0.16	0.00
Tg11g02730	0.00	1.15	0.00	0.00	0.00	0.00	0.00	0.00	0.00	0.00	0.00	0.00
Tg11g14670	0.00	0.18	0.00	0.00	0.00	0.00	0.00	0.00	0.00	0.00	0.00	0.00
Tg01g08850	0.62	2.18	0.80	0.00	0.00	0.00	0.00	0.00	0.22	0.00	0.00	0.00
Tg09g15630	2.41	6.26	0.09	0.22	0.00	0.00	0.00	0.00	0.54	0.00	0.00	0.00
Tg02g10510	0.84	3.40	0.00	0.00	0.00	0.00	0.00	0.00	0.00	0.00	0.00	0.00
Tg11g12110	0.17	0.76	0.11	0.00	0.00	0.00	0.00	0.00	0.00	0.00	0.00	0.00
Tg11g05530	0.30	4.63	0.00	2.27	0.18	0.00	0.00	0.00	0.19	0.00	0.00	0.00
Tg11g04450	0.47	2.27	0.00	0.66	0.00	0.00	0.00	0.00	1.40	0.00	0.00	0.00
Tg02g02730	12.30	66.67	26.91	39.17	0.32	0.38	0.29	0.32	33.50	2.32	1.96	4.53
Tg14g11050	0.53	2.50	0.46	0.71	1.62	1.98	0.82	1.34	14.71	15.34	4.46	5.10
TgUn272g00030	0.76	0.54	0.00	0.00	0.25	0.21	1.13	0.00	2.13	1.85	0.00	0.13
Tg11g07580	9.67	18.14	15.66	23.50	14.74	20.90	29.76	14.71	87.53	19.41	27.98	27.64
Tg01g12810	0.64	2.50	4.57	2.88	1.01	0.20	1.77	2.48	23.14	2.52	4.37	6.31
Tg02g05550	0.31	1.82	1.98	2.97	0.20	0.08	1.86	0.60	9.05	0.79	1.43	2.46
Tg05g10730	5.88	3.58	0.59	3.67	0.95	1.70	1.76	0.24	13.45	2.91	0.25	0.28
Tg11g11960	53.41	57.75	10.13	21.68	0.88	0.00	1.20	2.84	296.03	3.42	4.49	5.63
Tg02g09660	26.62	23.09	4.39	53.19	0.37	0.18	0.00	0.00	169.37	0.39	2.22	3.04
Tg02g09900	5.91	12.81	3.11	1.00	0.35	0.99	0.65	0.00	21.72	0.15	0.57	0.75
Tg15g11030	8.85	112.13	2.99	6.82	16.74	23.20	27.62	7.59	248.02	56.38	11.24	7.56
Tg18g09420	8.12	20.44	36.26	28.97	7.26	5.31	9.81	12.54	30.17	10.72	14.60	13.61
Tg12g03030	34.60	66.63	70.97	67.51	20.75	20.03	25.90	22.35	67.57	20.67	25.46	27.54
Tg10g05660	10.62	48.13	22.42	39.82	0.15	0.48	0.84	0.30	87.41	0.39	0.67	0.66
Tg18g04440	13.25	45.66	27.07	24.54	2.14	1.25	4.39	6.94	52.08	6.22	4.80	6.72
Tg08g06620	9.27	15.04	12.16	14.47	5.90	6.76	9.33	11.38	20.15	13.32	9.65	10.84
Tg12g03010	47.12	60.89	61.95	55.07	25.81	21.35	34.92	48.40	108.10	62.95	36.70	31.55
Tg07g01830	5.55	10.16	3.77	10.84	4.79	3.61	6.04	7.19	8.18	10.39	5.54	5.56
Tg05g19210	0.00	0.20	0.00	0.60	0.00	0.00	0.00	0.00	0.45	0.00	0.00	0.00

Tg07g01840	12.28	45.52	17.02	51.50	6.43	5.72	6.95	7.36	52.87	4.95	9.01	9.70
Tg10g01380	0.87	0.99	1.09	2.89	0.00	0.00	0.00	0.00	1.57	1.25	0.35	0.38
Tg17g02630	1.18	1.52	2.35	17.84	0.18	0.00	0.67	0.18	5.20	0.47	0.10	0.45
Tg02g15550	0.82	5.06	3.10	29.42	0.45	0.24	2.62	5.16	13.37	4.10	5.50	6.73
Tg17g08380	1.47	2.98	0.77	73.93	0.91	0.87	2.88	2.01	42.10	1.10	2.41	2.11
Tg06g07710	0.00	0.00	0.15	0.09	0.16	0.00	0.00	0.00	0.00	0.00	0.00	0.00
Tg05g12200	1.85	0.49	9.90	0.41	1.15	0.00	0.00	0.49	0.34	0.00	0.00	0.00
Tg18g11300	8.38	5.31	49.29	6.23	0.00	0.00	0.00	0.00	3.05	0.00	0.00	0.14
Tg13g09540	0.10	0.41	2.74	0.63	0.00	0.00	0.00	0.00	0.00	0.00	0.00	0.00
Tg06g07740	0.00	0.16	0.62	0.18	0.00	0.00	0.00	0.00	0.00	0.00	0.00	0.00
Tg09g03360	20.17	155.52	640.42	14.49	3.65	1.83	1.25	1.91	13.88	3.74	3.52	3.10
Tg14g02200	0.90	31.92	114.66	0.45	0.00	0.21	0.00	0.00	1.11	0.00	0.14	0.16
Tg06g17630	15.48	57.89	329.98	38.15	19.40	15.43	20.15	20.18	52.79	26.55	27.95	27.50
Tg08g17690	3.68	10.42	63.94	6.65	0.00	0.54	0.21	0.00	4.57	0.00	0.12	0.41
Tg07g09430	38.58	208.89	1150.97	18.87	6.21	7.76	3.38	3.34	52.09	11.76	6.21	7.45
Tg08g06610	27.56	63.79	108.05	20.41	11.40	8.33	20.05	17.99	42.23	12.76	18.79	18.31
Tg09g04510	1.24	2.04	3.40	1.59	0.00	0.00	0.00	0.00	0.00	0.00	0.00	0.11
Tg03g08530	138.50	155.50	444.30	127.98	10.76	9.71	12.97	15.67	80.78	23.05	14.06	20.71
Tg14g03980	0.00	0.00	4.79	4.80	1.81	0.00	0.00	0.00	0.00	0.00	0.00	0.00
Tg06g16210	9.88	13.62	31.56	14.82	0.43	0.17	0.79	1.30	22.27	2.03	11.19	15.95
Tg07g12160	0.72	0.43	1.62	0.50	0.00	0.00	0.00	0.00	1.56	0.12	0.67	0.42
Tg16g09090	11.40	2.79	26.90	6.19	0.00	0.00	0.00	0.00	26.80	0.43	3.75	4.25
Tg17g08390	42.81	44.32	266.29	72.71	1.72	2.10	3.88	5.35	148.93	11.15	15.37	19.90
Tg13g07150	46.39	13.61	310.16	36.67	21.62	18.72	25.92	40.52	187.71	52.85	53.62	57.24
Tg09g15920	35.20	16.60	18.58	71.84	10.58	10.36	20.71	44.33	32.06	56.84	54.08	62.53
Tg16g09080	1.85	2.67	2.24	3.42	0.00	0.00	0.00	0.00	1.61	0.68	1.39	5.07
Tg16g07170	1.40	2.56	3.33	3.26	0.00	0.00	0.00	0.00	0.20	0.16	0.00	0.47
Tg10g07670	1.18	1.94	1.51	2.97	0.00	0.00	0.00	0.00	0.00	0.00	0.00	0.00
Tg15g08390	1.27	1.48	1.82	2.79	0.00	0.00	0.00	0.00	0.00	0.00	0.00	0.08
Tg18g00740	0.40	1.28	0.75	2.09	0.00	0.00	0.00	0.00	0.26	0.00	0.00	0.00

Tg11g03710	1.81	4.75	1.25	7.62	0.00	0.00	0.00	0.18	2.10	0.00	0.10	0.23
Tg03g10560	0.11	0.47	0.00	0.45	0.00	0.00	0.00	0.00	0.00	0.00	0.00	0.00
Tg11g10750	15.08	29.03	10.07	28.36	3.53	2.23	5.07	7.62	5.67	3.95	4.14	5.87
Tg08g07120	0.08	0.00	0.21	1.73	0.42	0.00	0.39	0.00	0.00	0.00	0.11	0.00
TgUn720g00010	0.00	0.00	0.00	14.01	2.03	0.00	1.59	0.00	0.00	0.00	0.00	0.00
Tg17g09060	0.18	0.48	0.11	4.81	0.00	0.00	0.00	0.00	0.00	0.00	0.13	0.29
Tg15g11670	0.00	0.00	0.00	0.71	0.00	0.00	0.00	0.00	0.00	0.00	0.00	0.00
Tg12g02970	0.00	0.00	0.00	0.16	0.00	0.00	0.00	0.00	0.00	0.00	0.00	0.00
Tg01g11480	0.00	0.00	0.00	0.11	0.00	0.00	0.00	0.00	0.00	0.00	0.00	0.00
g10g06080	0.00	0.00	0.00	1.37	0.00	0.00	0.00	0.00	0.00	0.00	0.00	0.00
Tg12g08930	0.33	3.83	0.80	45.32	1.61	1.15	0.38	0.41	0.43	0.35	0.92	0.76
Tg11g07410	0.71	2.30	0.32	41.64	0.00	0.14	0.00	0.00	1.25	0.00	0.26	0.70
Tg13g06720	0.85	2.86	0.29	14.12	0.00	0.00	0.68	0.48	0.26	0.00	0.00	0.00
Tg15g02070	1.15	4.34	1.11	16.57	0.87	1.53	0.80	0.69	2.19	2.87	1.07	1.32
Tg05g19290	0.00	0.75	0.00	2.49	0.00	0.14	0.00	0.00	0.19	0.00	0.00	0.00
Tg15g01040	0.41	0.09	0.17	0.54	0.00	0.00	0.00	0.00	0.00	0.15	0.00	0.42
Tg02g10410	1.24	0.23	0.11	1.12	0.00	0.00	0.00	0.00	0.00	0.00	0.00	0.00
Tg16g13420	0.13	0.00	0.00	0.21	0.00	0.00	0.00	0.00	0.00	0.00	0.00	0.00

Sequence Read Archive (SRA) codigs of RNAseq samples used; [a]seedlings_SRR2080097; [b]roots_SRR2080096; [c]leaves_SRR2080095; [d]flowers_SRR2080094; [e]stem12yr1_SRR2080131; [f]stem12yr2_SRR2080132; [g]stem60yr1_SRR2080137; [h]stem60yr2_SRR2080138 [i]branch12yr1_SRR2080148; [j]branch12yr2_SRR2080149; [k]branch60yr1_SRR2080150; [l]branch60yr2_SRR2080151.

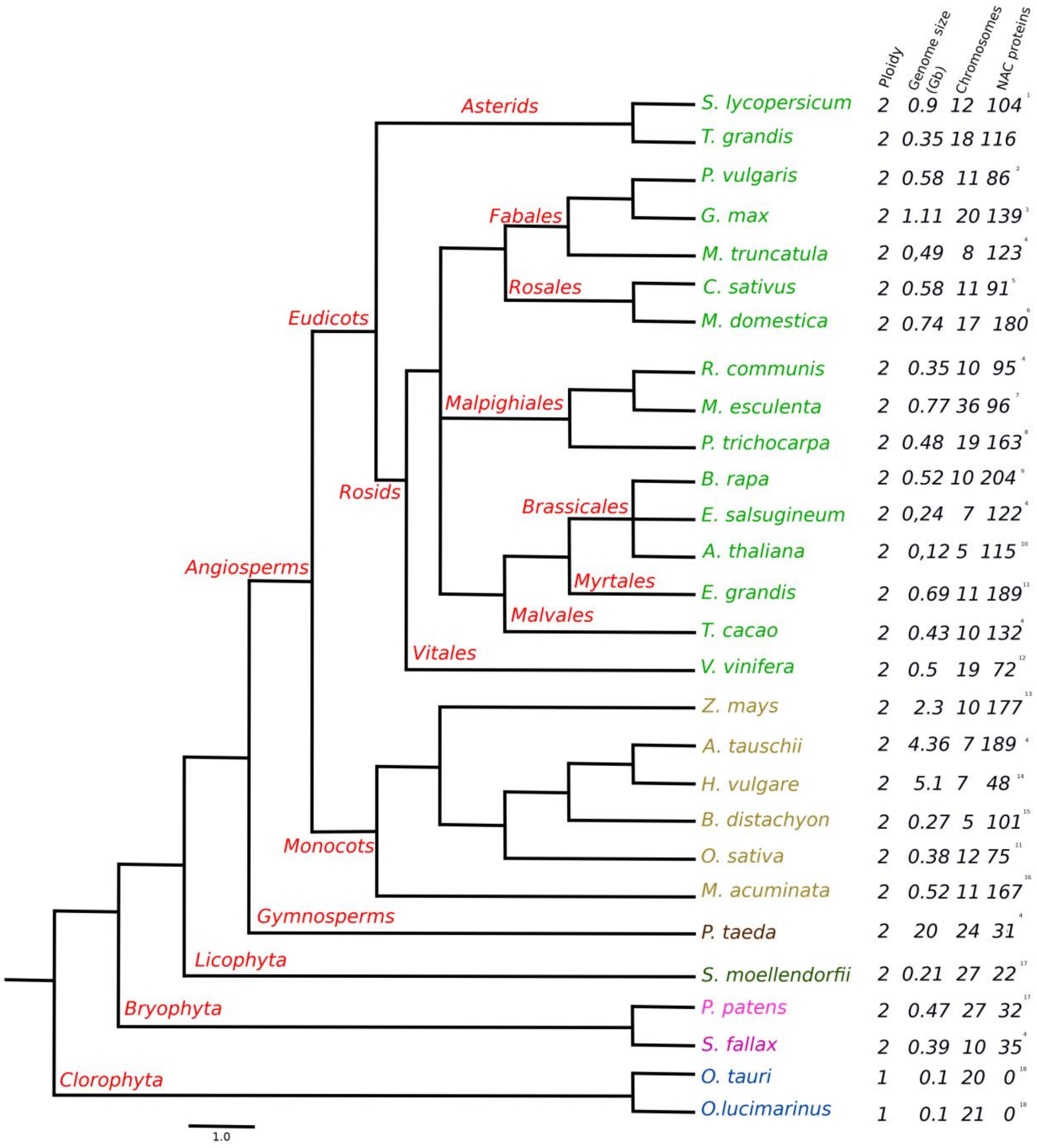

Figure S1. Phylogenetic profile of 28 plant species and numbers of identified NAC proteins. Ploidy level, the plant genome size (Gb), the number of chromosomes and total number of proteins identified in each genome. The genomes were annotated and assembled in Phytozome12 (https://phytozome.jgi.doe.gov). Subscripts on NAC proteins number refers to the Genome Reference of each species. The references are presented below.

1. Su, H., Zhang, S., Yin, Y. et al. Genome-wide analysis of NAM-ATAF1,2-CUC2 transcription factor family in Solanum lycopersicum. J. Plant Biochem. Biotechnol. 2015, 24, 176. https://doi.org/10.1007/s13562-014-0255-9
2. Wu, J., Wang, L. & Wang, S. Comprehensive analysis and discovery of drought-related NAC transcription factors in common bean. BMC Plant Biol, 2016, 16, 193. doi:10.1186/s12870-016-0882-5
3. Hussain, R.M., Ali, M., Feng, X. et al. The essence of NAC gene family to the cultivation of drought-resistant soybean (Glycine max L. Merr.) cultivars. BMC Plant Biol 2017, 17, 55. doi:10.1186/s12870-017-1001-y
4. Jin, J.P.; Tian, F.; Yang, D.C.; Meng, Y.Q.; Kong, L.; Luo, J.C.; Gao, G. PlantTFDB 4.0: toward a central hub for transcription factors and regulatory interactions in plants. Nucleic Acids Res. 2017, 45, 1040-1045. DOI: 10.1093/nar/gkw982.
5. Liu, X., wang, T., Bartholomew, E. et al. Comprehensive analysis of NAC transcription factors and their expression during fruit spine development in cucumber (Cucumis sativus L.). Hortic Res 2018, 5, 31. doi:10.1038/s41438-018-0036-z
6. H.Y. Su, S.Z. Zhang, X.W. Yuan, C.T. Chen, X.F. Wang, Y.J. Hao. Genome-wide analysis and identification of stress-responsive genes of the NAM–ATAF1, 2–CUC2 transcription factor family in apple. Plant Physiol. Biochem. 2013, 71 , pp. 11-21
7. Hu W, Wei Y, Xia Z, Yan Y, Hou X, et al. Genome-Wide Identification and Expression Analysis of the NAC Transcription Factor Family in Cassava. PLOS ONE, 2015, 10, e0136993. https://doi.org/10.1371/journal.pone.0136993

8. Hu R.; Guang, Q.; Yingzhen, K.; Dejing, K.; Qian, G.; Gongke, Z. Comprehensive Analysis of NAC Domain Transcription Factor Gene Family in Populus trichocarpa. BMC Plant Biol. 2010, 10, 145:1-145:23. DOI: 10.1186 / 1471-2229-10-145.
9. Liu, T., Song, X., Duan, W. et al. Genome-Wide Analysis and Expression Patterns of NAC Transcription Factor Family Under Different Developmental Stages and Abiotic Stresses in Chinese Cabbage Plant Mol Biol Rep, 2014, 32, 1041. https://doi.org/10.1007/s11105-014-0712-6
10. Ooka H, Satoh K, Doi K, Nagata T, Otomo Y, Murakami K, Matsubara K, Osato N, Kawai J, Carninci P,Hayashizaki Y, Suzuki K, Kojima K, Takahara Y, Yamamoto K, Kikuchi S: Comprehensive analysis of NAC family genes in Oryza sativa and Arabidopsis thaliana. DNA Res, 2003, 10, 239-247. DOI: 10.1093/dnares/10.6.239
11. Hussey, S.G., Saïdi, M.N., Hefer, C.A., Myburg, A.A. and Grima-Pettenati, J. Structural, evolutionary and functional analysis of the NAC domain protein family in Eucalyptus. New Phytol, 2015, 206: 1337-1350.
12. Wang, N., Zheng, Y., Xin, H. et al. Comprehensive analysis of NAC domain transcription factor gene family in Vitis vinifera Plant Cell Rep, 2013, 32: 61. https://doi.org/10.1007/s00299-012-1340-y
13. Li, Liang & Ma, Yiwen & Zhang, Shihuang & Hao, Zhuanfang & Li, Xinhai. Zea mays NAC transcription factor family members: Their genomic characteristics and relationship with drought stress. Research Journal of Biotechnology. 2015, 10. 63-77.
14. You J, Zhang L, Song B, Qi X, Chan Z. Systematic Analysis and Identification of Stress-Responsive Genes of the NAC Gene Family in Brachypodium distachyon. PLoS ONE, 2015, 10: e0122027. https://doi.org/10.1371/journal.pone.0122027
15. Christiansen, M.W., Holm, P.B. & Gregersen, P.L. Characterization of barley (Hordeum vulgare L.) NAC transcription factors suggests conserved functions compared to both monocots and dicots. BMC Res Notes, 2011, 4, 302. doi:10.1186/1756-0500-4-302
16. Cenci, A., Guignon, V., Roux, N. et al. Genomic analysis of NAC transcription factors in banana (Musa acuminata) and definition of NAC orthologous groups for monocots and dicotsPlant Mol Biol, 2014, 85: 63. https://doi.org/10.1007/s11103-013-0169-2
17. Aude Maugarny-Calès, Beatriz Gonçalves, Stefan Jouannic, Michael Melkonian, Gane Ka-Shu Wong, Patrick Laufs, Apparition of the NAC Transcription Factors Predates the Emergence of Land Plants, Molecular Plant, 2016, 9, 1345-1348. doi: https://doi.org/10.1016/j.molp.2016.05.016.
18. Zhu, T., Nevo, E., Sun, D. and Peng, J. PHYLOGENETIC ANALYSES UNRAVEL THE EVOLUTIONARY HISTORY OF NAC PROTEINS IN PLANTS. Evolution, 2012, 66: 1833-1848. doi:10.1111/j.1558-5646.2011.01553.x
