# Supplementary figures and images for: Analysis of NAC Domain Transcription Factor Genes of Tectona grandis L.f. Involved in Secondary Cell Wall Deposition
Source: Genes (Basel). 2019 Dec 23;11(1):20. doi: 10.3390/genes11010020 (PMC7016782; doi:10.3390/genes11010020)

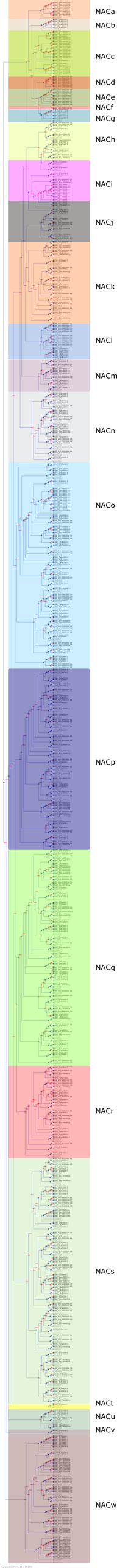

Supplement: Supplementary file 1 [file genes-11-00020-s001.zip › Figure S2.pdf]
